# Interacting Particle Solutions of Fokker–Planck Equations Through Gradient–Log–Density Estimation

**DOI:** 10.3390/e22080802

**Published:** 2020-07-22

**Authors:** Dimitra Maoutsa, Sebastian Reich, Manfred Opper

**Affiliations:** 1 Artificial Intelligence Group, Technische Universität Berlin, Marchstraße 23, 10587 Berlin, Germany; 2 Institute of Mathematics, University of Potsdam, Karl-Liebknecht-Str. 24/25, 14476 Potsdam, Germany; sereich@uni-potsdam.de

**Keywords:** stochastic systems, Fokker-Planck equation, interacting particles, multiplicative noise, gradient flow, stochastic differential equations

## Abstract

Fokker–Planck equations are extensively employed in various scientific fields as they characterise the behaviour of stochastic systems at the level of probability density functions. Although broadly used, they allow for analytical treatment only in limited settings, and often it is inevitable to resort to numerical solutions. Here, we develop a computational approach for simulating the time evolution of Fokker–Planck solutions in terms of a mean field limit of an interacting particle system. The interactions between particles are determined by the gradient of the logarithm of the particle density, approximated here by a novel statistical estimator. The performance of our method shows promising results, with more accurate and less fluctuating statistics compared to direct stochastic simulations of comparable particle number. Taken together, our framework allows for effortless and reliable particle-based simulations of Fokker–Planck equations in low and moderate dimensions. The proposed gradient–log–density estimator is also of independent interest, for example, in the context of optimal control.

## 1. Introduction

Many biological and physical systems are characterised by the presence of stochastic forces that influence their dynamics. These forces may be attributed either to intrinsic or extrinsic sources [1,2,3], i.e., arising either from random fluctuations of constituent subsystems [4,5], or from fluctuating interactions with the environment [6,7].

Often, deterministic analysis of these systems suffices to describe their macroscopic behaviour, and the fluctuations contribute only negligible perturbations around the deterministic dynamics. In systems biology, for example, rate equations describing mean concentrations of considered system’s species have provided a useful description of chemical reaction networks’ dynamics, and have enabled answering invaluable questions pertaining chemical systems comprising large numbers of molecules [8].

However, in several settings, the effect of stochastic forces influences considerably the resulting system’s behaviour by qualitatively altering its evolution. In those settings, random fluctuations have to be accounted for, and thus stochastic analysis becomes essential [9,10]. Phenomena such as stochastic resonance [11,12], noise induced transitions [13,14,15], and stochastic synchronisation, to name a few, are prevalent in many physical systems, and highlight the importance of considering fluctuating forces in the analysis of a system’s behaviour. Manifestations of these phenomena abound in nature and have been encountered in genetics [16], neuroscience [17,18], climate science [12,19,20] and other fields.

Indispensable tools for the analysis of stochastic systems are Kolmogorov equations, and in particular the Fokker–Planck equation (FPE) [21,22]. FPEs characterise the evolution of the probability density functions (PDF) for the state variables of dynamical systems described by stochastic differential equations (SDE). SDEs commonly arise in modeling random effects in systems with continuous state variables [23], or after diffusion approximation of Master equations for systems involving discrete state transitions in time [8,24,25]. The associated FPEs have been widely used for modelling stochastic phenomena in various fields, such as, for example, in physics, finance, biology, neuroscience, traffic flow [26].

Yet, although commonly used, explicit closed-form solutions of FPEs are rarely available [27], especially in settings where the underlying dynamics is nonlinear. In particular, exact analytic solutions may be obtained only for a restricted class of systems following linear dynamics perturbed by white Gaussian noise, and for some nonlinear Hamiltonian systems [21,28]. Further systems that admit analytical treatment independent of system dimension are those with discrete state transitions approximated via van Kampen expansion (linear noise approximation), resulting thus in linear SDEs with time dependent coefficients [9].

Existing numerical approaches for computing Fokker–Planck solutions may be grouped into three broad categories: grid-based, semi-analytical, and sample-based methods. The first category comprises mainly finite difference and finite element methods [29,30,31,32]. These frameworks, based on integration of FPEs employing numerical solvers for partial differential equations, entail computationally demanding calculations with inherent finite spatial resolution [33].

Conversely, semi-analytical approaches try to reduce the number of required computations by assuming conditional Gaussian structures [34], or by employing cumulant neglect closures [35], statistical linearisation [36,37], or stochastic averaging [38]. Although efficient in the settings they are devised for, their applicability is limited since the resulting solutions are imprecise or unstable in certain settings.

On the other hand, in the sample-based category, Monte Carlo methods resort to stochastic integration of a large number of *independent* stochastic trajectories that as an ensemble represent the probability density [39,40]. These methods are appropriate for computing *unbiased* estimates of exact expectations from empirical averages. Nevertheless, as we show in the following, cumulants of resulting distributions exhibit strong temporal fluctuations when the number of simulated trajectories is not sufficiently large.

Surprisingly, there is an alternative sample-based approach built on *deterministic* particle dynamics. In this setting, the particles are not independent, but they rather *interact* via an (approximated) probability density, and the FPE describes the mean field limit when their number grows to infinity. This approach introduces a bias in the approximated expectations, but significantly reduces the variance for a given particle number.

Recent research, see e.g., [41,42,43,44], has focused on particle methods for models of thermal equilibrium, where the stationary density is known analytically. For these models, interacting particle methods have found interesting new applications in the field of probabilistic Bayesian inference: by treating the Bayesian posterior probability density as the stationary density of a FPE, the particle dynamics provides posterior samples in the long time limit. For this approach, the particle dynamics are constructed by exploiting the gradient structure of the probability flow of the FPE. This involves the relative entropy distance to the equilibrium density as a Lyapunov function. Unfortunately, this structure does not apply to general FPEs in *non–equilibrium* settings, where the stationary density is usually unknown.

In this article, we introduce a framework for interacting particle systems that may be applied to general types of Fokker–Planck equations. Our approach is based on the fact that the instantaneous effective force on a particle due to diffusion is proportional to the *gradient* of the *logarithm* of the exact probability *density* (GLD). Rather than computing a differentiable estimate of this density (say by a kernel density estimator), we estimate the GLD directly without requiring knowledge of a stationary density. Therefore, we introduce an approximation to the effective force acting on each particle, which becomes exact in the large particle number limit given the consistency of the estimator.

Our approach is motivated by recent developments in the field of machine learning, where GLD estimators have been studied independently and are used to fit probabilistic models to data. An application of these techniques to particle approximations for FPE is, to our knowledge, new. (The approach in [45] uses a GLD estimator different from ours for particle dynamics but with a probability flow towards equilibrium which is not given by a standard FPE.) Furthermore, our method provides straightforward approximations of entropy production rates, which are of primary importance in non–equilibrium statistical physics [46].

This article is organised as follows: Section 2 describes the deterministic particle formulation of the Fokker–Planck equation. Section 3 shows how a gradient of the logarithm of a density may be represented as the solution of a variational problem, while in Section 4 we discuss an empirical approximation of the gradient-log-density. In Section 5, we introduce function classes for which the variational problem may be solved explicitly, while in Section 6 we compare the temporal derivative of empirical expectations based on the particle dynamics with exact results derived from the Fokker–Planck equation. Section 7 is devoted to the class of equilibrium Fokker–Planck equations, where we discuss relations to Stein Variational Gradient Descent and other particle approximations of Fokker–Planck solutions. In Section 8, we show how our method may be extended to general diffusion processes with state-dependent diffusion, while Section 9 discusses how our framework may be employed to simulate second order Langevin dynamics. In Section 10 we demonstrate various aspects of our method by simulating Fokker–Planck solutions for different dynamical models. Finally, we conclude with a discussion and an outlook in Section 11.

## 2. Deterministic Particle Dynamics for Fokker–Planck Equations

We consider Fokker–Planck equations of the type

(1) $$\frac{\partial p_{t}\left(x\right)}{\partial t}=-\nabla \cdot \left[f\left(x\right)p_{t}\left(x\right)-\frac{\sigma^{2}}{2}\nabla p_{t}\left(x\right)\right].$$

Given an initial condition $p_{0}\left(x\right)$, Equation (1) describes the temporal development of the density $p_{t}\left(x\right)$ for the random variable $X\left(t\right)\in R^{d}$ following the stochastic differential equation

(2) dX(t)=f(X(t))dt+σdB(t).

In Equation (2), $f\left(x\right)\in R^{d}$ denotes the drift function characterising the deterministic part of the driving force, while $dB\left(t\right)\in R^{d}$ represents the differential of a vector of independent Wiener processes capturing stochastic, Gaussian white noise excitations. For the moment, we restrict ourselves to state independent and diagonal *diffusion matrices*, i.e., diffusion matrices independent of X(t) (additive noise) with diagonal elements $\sigma^{2}$ characterising the noise amplitude in each dimension. Extensions to more general settings are deferred to Section 8.

We may rewrite the FPE Equation (1) in the form of a *Liouville* equation

(3) $$\frac{\partial p_{t}\left(x\right)}{\partial t}=-\nabla \cdot \left[g\left(x,t\right)\;p_{t}\left(x\right)\right]$$

for the *deterministic* dynamical system

(4) $$\frac{dX}{dt}=g\left(X,t\right)\;,\;X\left(0\right)∼p_{0}\left(x\right),$$

(dropping the time argument in X(t) for simplicity) with velocity field

(5) $$g\left(x,t\right)=f\left(x\right)-\frac{\sigma^{2}}{2}\nabla \ln p_{t}\left(x\right)\;.$$

Hence, by evolving an ensemble of *N* *independent* realisations of Equation (4) (to be called ’particles’ in the following) according to

(6) $$\frac{dX_{i}}{dt}=g\left(X_{i},t\right)\;,\;i=1,\ldots ,N\;X_{i}\left(0\right)∼p_{0}\left(x\right),$$

we obtain an empirical approximation to the density $p_{t}\left(x\right)$.

Since the only source of randomness in Equation (4) can be attributed to the initial conditions $X_{i}\left(0\right)$, averages computed from the particle approximation (Equation (6)) are expected to have smaller variance compared to *N* independent realisations of the SDE (Equation (2)). Unfortunately, this approach requires perfect knowledge of the unknown instantaneous density $p_{t}\left(x\right)$ (Equation (5)), which is actually the unknown quantity of interest.

Here, we circumvent this issue by introducing *statistical estimators* for the term $\nabla \ln p_{t}\left(x\right)$, computed from the entire ensemble $\left(X_{1}\left(t\right)\right),\ldots ,X_{N}\left(t\right))$ of particles at time *t*. Although this additional approximation introduces interactions among the particles via the estimator, for sufficiently large particle number *N*, fluctuations of the estimator are expected to be negligible and the limiting dynamics should converge to its mean field limit (Equation (4)) provided the estimator is asymptotically consistent. Thus, rather than computing a differentiable approximation to $p_{t}\left(x\right)$ from the particles, e.g., by a kernel density estimator, we show in the following section, how the function $\nabla \ln p_{t}\left(x\right)$ may be directly estimated from samples of $p_{t}\left(x\right)$.

## 3. Variational Representation of Gradient–Log–Densities

To construct a gradient–log–density (GLD) estimator we rely on a variational representation introduced by *Hyvärinen* in his *score–matching* approach for the estimation of non–normalised statistical models [47]. We favoured this approach over other estimators [48,49] due to its flexibility to adapt to different function classes chosen to approximate the GLD.

Here, we use a slightly more general representation compared to [47] allowing for an extra arbitrary reference function $r\left(x\right)=(r^{\left(1\right)}\left(x\right),\ldots ,r^{\left(d\right)}\left(x\right))$ such that the component α of the gradient is represented as

(7) $$\partial_{\alpha }\ln p\left(x\right)=r^{\left(\alpha \right)}\left(x\right)+\arg\min_{\phi }\;L_{\alpha }^{r}\left[\phi ,p\right]\left(x\right),$$

where $\partial_{\alpha }≐\frac{\partial }{\partial x^{\left(\alpha \right)}}$ stands for the partial derivative with respect to coordinate α of the vector $x\equiv (x^{\left(1\right)},\ldots x^{\left(d\right)})$.

The cost function is defined as an expectation with respect to the density p(x) by

(8) $$L_{\alpha }^{r}\left[\phi ,p\right]=\int p\left(x\right)\left(\phi^{2}\left(x\right)+2r^{\left(\alpha \right)}\left(x\right)\phi \left(x\right)+2\partial_{\alpha }\phi \left(x\right)\right)dx,$$

with dx representing the volume element in $R^{d}$. To obtain this relation, we use integration by parts (assuming appropriate behaviour of densities and ϕ at boundaries), and get

(9) $$\begin{array}{c} L_{\alpha }^{r}\left[\phi ,p\right]=\int p\left(x\right){\left(\phi \left(x\right)+r^{\left(\alpha \right)}\left(x\right)-\partial_{\alpha }\ln p\left(x\right)\right)}^{2}dx-\int p\left(x\right){\left(\partial_{\alpha }\ln p\left(x\right)-r^{\left(\alpha \right)}\left(x\right)\right)}^{2}dx. \end{array}$$

Minimisation with respect to ϕ yields Equation (7).

## 4. Gradient–Log–Density Estimator

To transform the variational formulation into a GLD estimator based on *N* sample points $\left(X_{1},\ldots ,X_{N}\right)$, we replace the density p(x) in Equation (8) by the empirical distribution ${\hat{p}}_{t}\left(x\right)=\frac{1}{N}\sum_{i=1}^{N}\delta \left(x-X_{i}\left(t\right)\right)$, i.e.,

(10) $$L_{\alpha }^{r}\left[\phi ,p_{t}\right]\approx L_{\alpha }^{r}\left[\phi ,{\hat{p}}_{t}\right]=\frac{1}{N}\sum_{i=1}^{N}\left(\phi^{2}\left(X_{i}\right)+2r^{\left(\alpha \right)}\left(X_{i}\right)\phi \left(X_{i}\right)+2\partial_{\alpha }\phi \left(X_{i}\right)\right),$$

and

(11) $$\partial_{\alpha }\ln p_{t}\left(x\right)\approx r^{\left(\alpha \right)}\left(x\right)+\arg\min_{\phi \in F}\;L_{\alpha }^{r}\left[\phi ,{\hat{p}}_{t}\right]\left(x\right),$$

where F is an appropriately chosen family of functions with controllable complexity. By introducing the estimator of Equation (11) in Equation (6), we obtain a particle representation for the Fokker–Planck equation

(12) $$\frac{dX_{i}^{\left(\alpha \right)}}{dt}=f^{\left(\alpha \right)}\left(X_{i}\right)-\frac{\sigma^{2}}{2}\left(r^{\left(\alpha \right)}\left(X_{i}\right)+\arg\min_{\phi \in F}\;L_{\alpha }^{r}\left[\phi ,{\hat{p}}_{t}\right]\left(X_{i}\right)\right),$$

for i=1,…,Nandα=1,…,d, with

$${\hat{p}}_{t}\left(x\right)=\frac{1}{N}\sum_{i=1}^{N}\delta \left(x-X_{i}\right)\;,\;X_{i}\left(0\right)∼p_{0}\left(x\right).$$

Although in this article, we use reference functions r(·)≡0 for all simulated examples, the choice $r\left(x\right)=\frac{2}{\sigma^{2}}f\left(x\right)$, which cancels the first two terms in Equation (12), leads to interesting relations with other particle approaches for simulating Fokker–Planck solutions for equilibrium systems (c.f. Section 7) and impacts on the numerical approximation of ϕ (c.f. Section 5). In particular, when considering Brownian dynamics, where f(x)=−∇U(x), the choice $r\left(x\right)=-\frac{2}{\sigma^{2}}\nabla U\left(x\right)$ leads to ϕ(x)=0 once the system has reached thermal equilibrium. More generally speaking, one may choose $r\left(x\right)=\frac{2}{\sigma^{2}}\nabla \ln p_{*}$, where $p_{*}$ is an appropriate reference measure such as the equilibrium measure of the underlying stochastic process.

### Estimating the Entropy Rate

Interestingly, the variational approach provides us with a simple, built in method for computing the entropy rate (temporal change of entropy) of the stochastic process (Equation (2)).

Using the FPE (1) and integration by parts, one can derive the well-known relation, see e.g., [50],

(13) $$-\frac{d}{dt}\int p_{t}\left(x\right)\ln p_{t}\left(x\right)dx=\frac{\sigma^{2}}{2}\sum_{\alpha =1}^{d}\int p_{t}\left(x\right){\left(\partial_{\alpha }\ln p_{t}\left(x\right)\right)}^{2}dx+\int p_{t}\left(x\right)\nabla \cdot f\left(x\right)dx.$$

The first term on the right hand side is usually called entropy production, whereas the second term corresponds to the entropy flux. In the stationary state, the total entropy rate vanishes. For equilibrium dynamics, both terms vanish individually at stationarity. This should be compared to the minimum of the cost function (Equation (9)), which for r≡0, equals

(14) $$\min_{\phi }L_{\alpha }^{0}\left[\phi ,p_{t}\right]=-\int p_{t}\left(x\right){\left(\partial_{\alpha }\ln p_{t}\left(x\right)\right)}^{2}dx.$$

Thus, we obtain the estimator

(15) $$-\frac{d}{dt}\int p_{t}\left(x\right)\ln p_{t}\left(x\right)dx\approx -\frac{\sigma^{2}}{2}\sum_{\alpha =1}^{d}\min_{\phi }L_{\alpha }^{0}\left[\phi ,{\hat{p}}_{t}\right]+\frac{1}{N}\sum_{i=1}^{N}\nabla \cdot f\left(X_{i}\right).$$

We will later see for the case of equilibrium dynamics that a similar method may be employed to approximate the relative entropy distance to the equilibrium density.

## 5. Function Classes

In the following, we discuss choices for families of functions F leading to explicit, closed form solutions for estimators.

### 5.1. Linear Models

A simple possibility is to choose linearly parametrised functions of the form

(16) $$\phi \left(x\right)=\sum_{k=1}^{m}a_{k}\phi_{k}\left(x\right)\;,$$

where the $\phi_{k}\left(x\right)$ are appropriate basis functions, e.g., polynomials, radial basis functions or trigonometric functions. For this linear parametrisation, the empirical cost (Equation (10)) is quadratic in the parameters $a_{k}$ and can be minimised explicitly. A straightforward computation from Equation (12) shows that

(17) $$\begin{array}{c} \frac{dX_{i}}{dt}=f\left(X_{i}\right)-\frac{\sigma^{2}}{2}r\left(X_{i}\right)+\frac{\sigma^{2}}{2}\sum_{k,j=1}^{m}{\left(C^{-1}\right)}_{kj}\phi_{k}\left(X_{i}\right)\sum_{l=1}^{N}\left\{\nabla \phi_{j}\left(X_{l}\right)+\phi_{j}\left(X_{l}\right)r\left(X_{l}\right)\right\}, \end{array}$$

with $C_{kl}=\sum_{i=1}^{N}\phi_{k}\left(X_{i}\right)\phi_{l}\left(X_{i}\right)$.

Here, we require the number of samples to be greater than the number of employed basis functions, i.e., N≥m+1, to have a non–singular matrix *C*. This restriction may be lifted by adding a penalty to the empirical cost (Equation (10)) for regularisation, similar to ridge regression estimators. Equation (17) is independent of the reference function *r*, when *r* belongs to the linear span of the selected basis functions. However, this model class with a finite parameter number has limited complexity.

When the particle number *N* grows large, we expect convergence of the dynamics to a mean field limit which would be given by

$$\begin{array}{c} \frac{dX_{i}}{dt}=f\left(X_{i}\right)-\frac{\sigma^{2}}{2}r\left(X_{i}\right)+\frac{\sigma^{2}}{2}\sum_{k,j=1}^{m}{\left(C^{-1}\right)}_{kj}\phi_{k}\left(X_{i}\right){\langle \nabla \phi_{j}\left(x\right)+\phi_{j}\left(x\right)r\left(x\right)\rangle }_{q_{t}}, \end{array}$$

where the brackets ${\langle \cdot \rangle }_{q_{t}}$ denote expectation with respect to the limiting density $q_{t}\left(x\right)$ and $C_{kl}={\langle \phi_{k}\left(X_{i}\right)\phi_{l}\left(X_{i}\right)\rangle }_{q_{t}}$. Since the linear model class (Equation (16)) exhibits limited complexity for fixed *m*, we do not expect the approximated solution $q_{t}\left(x\right)$ to equal the exact solution $p_{t}\left(x\right)$ of the FPE. Nevertheless, for rare cases, where both $\nabla \ln p_{t}\left(x\right)$ and also r(x) are linear combinations of the employed basis functions for all times *t*, $q_{t}\left(x\right)$ would provide an exact solution. For example, in a setting with linear drift function f(x)=−γx, reference function r(x)=0, and dimensionality d=1, a basis consisting of a constant $\phi_{1}\left(x\right)=1$ and a linear function $\phi_{2}\left(x\right)=x$, would be able to perfectly represent the GLD of the Gaussian density $p_{t}\left(x\right)$.

### 5.2. Kernel Approaches

Here, we consider a family F of functions for which the effective number of parameters to be computed is not fixed beforehand, but rather increases with the sample number *N*: a *reproducing kernel Hilbert space* (RKHS) of functions defined by a positive definite (Mercer) kernel K(·,·). Statistical models based on such function spaces have played a prominent role in the field of machine learning in recent years [51].

A common, kernel-based approach to regularise the minimisation of empirical cost functions is via penalisation using the RKHS norm ${∥\cdot ∥}_{\mathrm{RKHS}}$ of functions in F. This can also be understood as a penalised version of a linear model (Equation (16)) with infinitely many feature functions $\phi_{k}$. For so-called universal kernels [52], this unbounded complexity suggests that we could expect asymptotic convergence of the GLD estimator (see [53] for related results) and a corresponding convergence of the particle model to the FPE as its mean field limit. However, a rigorous proof may not be trivial, since particles in our setting are not independent.

The explicit form of the kernel-based approximation is given by

(18) $$\partial_{\alpha }\ln p\left(x\right)\approx r^{\left(\alpha \right)}\left(x\right)+\arg\min_{\phi \in F}\;\left\{L_{\alpha }^{r}\left[\phi ,\hat{p}\right]+\frac{\lambda }{N}{∥\phi ∥}_{\mathrm{RKHS}}^{2}\right\}\left(x\right),$$

where the parameter λ controls the strength of the penalisation. Again, this optimisation problem can be solved in closed form in terms of matrix inverses. One can prove a *representer theorem* which states that the minimiser ϕ(x) in Equation (18) is a linear combination of kernel functions evaluated at the sample points $X_{i}$, i.e.,

(19) $$\phi \left(x\right)=\sum_{i=1}^{N}a_{i}K\left(x,X_{i}\right).$$

For such functions, the RKHS norm is given by

(20) $${∥\phi ∥}_{\mathrm{RKHS}}^{2}=\sum_{i,j=1}^{N}a_{i}a_{j}K\left(X_{i},X_{j}\right).$$

Hence, this representation leads again to a quadratic form in the *N* coefficients.

The solution of the minimisation problem is given by

(21) $$a_{j}=-\sum_{k=1}^{N}{\left({\left(K^{2}+\lambda K\right)}^{-1}\right)}_{jk}\sum_{l=1}^{N}\left\{\partial_{\alpha_{l}}K\left(X_{l},X_{k}\right)+K\left(X_{l},X_{k}\right)r^{\left(\alpha \right)}\left(X_{l}\right)\right\},$$

where $K_{ij}≐K\left(X_{i},X_{j}\right)$. Similar approaches for kernel-based GLD estimators have been discussed in [48,49]. For r(·)=0, Equation (21) agrees with the GLD estimator of [48] derived by inverting *Stein’s* equation, or by minimising the *Kernelised Stein discrepancy*.

The resulting particle dynamics is given by

(22) $$\frac{dX_{i}}{dt}=f\left(X_{i}\right)-\frac{\sigma^{2}}{2}r\left(X_{i}\right)+\frac{\sigma^{2}}{2}\sum_{k=1}^{N}{\left({\left(K+\lambda I\right)}^{-1}\right)}_{ik}\sum_{l=1}^{N}\left\{\nabla_{l}K\left(X_{l},X_{k}\right)+K\left(X_{l},X_{k}\right)r\left(X_{l}\right)\right\}.$$

Please note that here the inverse matrix also depends on the particles $X_{k}$.

We may simplify Equation (22) by adding and subtracting a term $\lambda \delta_{kl}r\left(X_{l}\right)$ in the summation over *l*, with $\delta_{kl}$ denoting the Kronecker delta. This yields

(23) $$\frac{dX_{i}}{dt}=f\left(X_{i}\right)+\frac{\sigma^{2}}{2}\sum_{k=1}^{N}{\left({\left(K+\lambda I\right)}^{-1}\right)}_{ik}\left\{\sum_{l=1}^{N}\nabla_{l}K\left(X_{l},X_{k}\right)-\lambda r\left(X_{k}\right)\right\}.$$

In the limit of small λ, the right hand side becomes independent of the reference function *r*.

In the present article, we employ Gaussian radial basis function (RBF) kernels given by

(24) $$K\left(x,x^{\prime }\right)=\exp\left[-\frac{1}{2\;l^{2}}{∥x-x^{\prime }∥}^{2}\right],$$

with a length scale *l*. A different possibility would be given by kernels with a *finite dimensional* feature representation

(25) $$K\left(x,x^{\prime }\right)=\sum_{j=1}^{m}\phi_{j}\left(x\right)\phi_{j}\left(x^{\prime }\right),$$

which may also be interpreted as a *linear model* as in Equation (16) with a $L_{2}$ penalty on the unknown coefficients.

### 5.3. A Sparse Kernel Approximation

Since the inversions of the N×N matrices in Equation (22) have to be performed at each step of a time discretised ODE system (Equation (22)), for large particle number *N*, the cubic complexity becomes too computationally demanding. Hence, here, we resort to a well established approximation in machine learning to overcome this issue, by applying a sparse approximation to the optimisation problem of Equation (18), see e.g., [54]. In particular, we introduce a smaller set of M≪N*inducing points*${\left\{z_{k}\right\}}_{k=1}^{M}$ that need not necessarily be a subset of the *N* particles. We then minimise the penalised cost function (Equation (18)) in the finite dimensional family of functions

(26) $$\phi \left(x\right)=\sum_{i=1}^{M}a_{i}K\left(x,z_{i}\right).$$

This may also be understood as a special linear parametric approximation. To keep matrices well conditioned, in practice we add a small ’jitter’ term to Equation (18), i.e., we use

(27) $${\lambda ∥\phi ∥}_{\mathrm{RKHS}}^{2}+\epsilon {∥\phi ∥}_{2}^{2},$$

as the total penalty. In the limit λ,ϵ→0, this representation reduces to an approximation of the form of Equation (16) with *M* basis functions $K(\cdot ,z_{l})$ for l=1,…,M.

By introducing the matrices

(28) $$K_{kl}^{zz}≐K\left(z_{k},z_{l}\right)+\epsilon \delta_{kl},\;K_{ij}^{xz}≐K\left(X_{i},z_{j}\right),$$

and

(29) $$A≐K^{xz}{\left[\left(\lambda +\epsilon \right)I+{\left(K^{zz}\right)}^{-1}{\left(K^{xz}\right)}^{⊤}\left(K^{xz}\right)\right]}^{-1}{\left(K^{zz}\right)}^{-1},$$

we replace the particle dynamics of Equation (22) by

(30) $$\frac{dX_{i}}{dt}=f\left(X_{i}\right)-\frac{\sigma^{2}}{2}r\left(X_{i}\right)+\frac{\sigma^{2}}{2}\sum_{k}A_{ik}\sum_{l}\left\{\nabla_{l}K\left(X_{l},z_{k}\right)+K\left(X_{l},z_{k}\right)r\left(X_{l}\right)\right\}.$$

Hence, for this approximation we have to invert only M×M matrices. For fixed *M*, the complexity of the GLD estimator is limited. Results for log–density estimators in machine learning (obtained for independent data) indicate that for a moderate growth of the number of inducing points *M* with the number of particles *N*, similar approximation rates may be obtained as for full kernel approaches.

## 6. A Note on Expectations

In this section, we present a preliminary discussion of the quality of the particle method to approximate expectations of scalar functions *h* of the random variable X(t). We concentrate on the temporal development of h(X(t)). While it would be important to obtain an estimate of the approximation error over time, we will defer such an analysis to future publications and only concentrate on a result for the first time derivative of expectations, i.e., the evolution over infinitesimal times.

Using the FPE (Equation (1)) and integrations by parts, one derives the exact result

(31) $$\frac{d\langle h(X)\rangle }{dt}=\langle L_{x}h\left(X\right)\rangle ,$$

where 〈·〉 denotes the expectation with respect to $p_{t}\left(x\right)$ and the operator $L_{x}$ is defined as

(32) $$L_{x}≐f\left(X\right)\cdot \nabla +\frac{\sigma^{2}}{2}\nabla^{2}.$$

In Equation (32), $L_{x}$ denotes the *generator* of the diffusion process defined by the corresponding stochastic differential equation (Equation (2)). To obtain a related result for the particle dynamics and the empirical expectations denoted by ${\langle \cdot \rangle }_{{\hat{p}}_{t}}$, we employ the relation

(33) $$\frac{d{\langle h\left(X\right)\rangle }_{\hat{p}t}}{dt}={\langle \nabla h\left(X\right)\cdot \frac{dX}{dt}\rangle }_{\hat{p}t}=\frac{1}{N}\sum_{i=1}^{N}\nabla h\left(X_{i}\right)\cdot \frac{dX_{i}}{dt},$$

where we have used the chain rule for the time derivative.

We will focus initially on the dynamics based on basis functions. Expressing the sum over $X_{l}$ in Equation (17) as an expectation, we obtain

(34) $$\frac{dX_{i}}{dt}=f\left(X_{i}\right)-\frac{\sigma^{2}}{2}r\left(X_{i}\right)+\frac{N\sigma^{2}}{2}\sum_{k,j=1}^{m}{\left(C^{-1}\right)}_{kj}\phi_{k}\left(X_{i}\right){\langle \left(r(X)+\nabla \right)\phi_{j}\left(X\right)\rangle }_{\hat{p}t}.$$

Hence, by inserting Equation (34) into Equation (33) and adding and subtracting the term $\frac{\sigma^{2}}{2}{\langle \nabla^{2}h\left(X\right)\rangle }_{\hat{p}t}$ we obtain

(35) $$\frac{d{\langle h\left(X\right)\rangle }_{\hat{p}t}}{dt}={\langle L_{x}h\left(X\right)\rangle }_{\hat{p}t}+\Delta ,$$

where the remainder term is given by

(36) $$\Delta =\frac{\sigma^{2}}{2}{\langle \left(r(X)+\nabla \right)\cdot \left(\hat{\nabla }h\left(x\right)-\nabla h\left(x\right)\right)\rangle }_{\hat{p}t}.$$

For the approximation of the vectorial function ∇h(x) we have

(37) $$\hat{\nabla }h\left(x\right)=\sum_{j,k=1}^{m}\phi_{j}\left(x\right){\left(C^{-1}\right)}_{jk}\sum_{i=1}^{N}\phi_{k}\left(X_{i}\right)\nabla h\left(X_{i}\right).$$

A simple comparison shows that each component of the vector $\hat{\nabla }h\left(x\right)$ can be written as the minimiser of $\sum_{l=1}^{N}{\left(\Phi \left(X_{l}\right)-\partial_{\alpha }h\left(X_{l}\right)\right)}^{2}$ where Φ(x) is a linear combination of basis functions. Hence, $\hat{\nabla }h\left(x\right)$ equals the best approximation of the vectorial function ∇h(x) based on the ’data’ $\nabla h(X_{l})$ using regression with basis functions. Thus, if ∇h(x) is well approximated by basis functions, the remainder Δ is small. If indeed $\nabla h\left(x\right)=\sum_{n=1}^{M}c_{n}\phi_{n}\left(x\right)$, for some $c_{n}\in R^{d}$, the remainder term vanishes, Δ=0. By its similarity to the finite basis function model, this result should also be valid for the sparse kernel dynamics of Equation (30), when the penalty λ is small. One might conjecture that the temporal development of expectations for reasonably smooth functions might be faithfully represented by the particle dynamics. This conjecture is supported by our numerical results.

A similar result also holds for the dynamics of Equation (22). In this case, the function $\hat{\nabla }h\left(x\right)$ in the remainder Δ (Equation (36)) is given by

(38) $$\hat{\nabla }h\left(x\right)=\sum_{j,k=1}^{N}K\left(x,X_{j}\right){\left({\left(K+\lambda I\right)}^{-1}\right)}_{jk}\nabla h\left(X_{k}\right),$$

which has also an interpretation as an approximation of ∇h(x) by regularised kernel regression.

## 7. Equilibrium Dynamics

An important class of stochastic dynamical systems describe *thermal equilibrium*, for which the drift function *f* is the negative gradient of a potential *U*, while the limiting equilibrium density $p_{\infty }$ is explicitly given by a Gibbs distribution:

(39) $$\begin{array}{c} f(x)=-\nabla U(x) \end{array}$$

(40) $$\begin{array}{c} \nabla \ln p_{\infty }\left(x\right)=\frac{2}{\sigma^{2}}f\left(x\right). \end{array}$$

For this class of models, our method provides a simple and built-in estimator for the relative entropy between the instantaneous and the equilibrium density, $p_{t}$ and $p_{\infty }$ respectively. As we discuss here, our framework may also be related to two other particle approaches that converge to the (approximate) equilibrium density.

### 7.1. Relative Entropy

The relative entropy or *Kullback–Leibler divergence* is defined as

(41) $$D\left(p_{t}|p_{\infty }\right)≐\int p_{t}\left(x\right)\ln\frac{p_{t}\left(x\right)}{p_{\infty }\left(x\right)}dx.$$

Following a similar calculation that led to Equation (13), we obtain

(42) $$\begin{array}{c} \frac{d}{dt}D\left(p_{t}|p_{\infty }\right)=-\frac{\sigma^{2}}{2}\int p_{t}\left(x\right){∥\nabla \ln p_{t}\left(x\right)-\nabla \ln p_{\infty }\left(x\right)∥}^{2}dx=-\frac{2}{\sigma^{2}}\int p_{t}\left(x\right){∥g\left(x,t\right)∥}^{2}dx, \end{array}$$

where g(x,t) indicates the velocity field of the particle system defined in Equation (4). The first equality holds for arbitrary drift functions. To obtain the second equality, we have inserted the explicit result for $p_{\infty }$.

Hence, we may compute the relative entropy at any time *T* as a time integral

(43) $$D\left(p_{T}|p_{\infty }\right)=D\left(p_{0}|p_{\infty }\right)-\frac{2}{\sigma^{2}}\int_{0}^{T}\left\{\int p_{t}\left(x\right){∥g\left(x,t\right)∥}^{2}dx\right\}dt,$$

where the inner expectation is easily approximated by our particle algorithm. This result shows that the exact velocity field g(x,t) converges to 0 for t→∞, and one expects particles to also converge to fixed points. For other non–equilibrium systems, asymptotic fixed points are, however, the exception.

### 7.2. Relation to Stein Variational Gradient Descent

Recently, *Stein variational gradient descent* (SVGD), a kernel-based particle algorithm, has attracted considerable attention in the machine learning community [55,56]. The algorithm is designed to provide approximate samples from a given density $p_{\infty }$ as the asymptotic fixed points of a deterministic particle system. Setting $-\ln p_{\infty }\left(x\right)=U\left(x\right)+const$, SVGD is based on the dynamics

(44) $$\frac{dX_{i}}{dt}=\sum_{l}\left\{-K\left(X_{i},X_{l}\right)\nabla U\left(X_{l}\right)+\nabla_{l}K\left(X_{i},X_{l}\right)\right\}.$$

This may be compared to our approximate FPE dynamics (Equation (22)) for the equilibrium case by setting $\sigma^{2}=2$ and r(x)=f(x)=−∇U(x). For this setting, both algorithms have in fact, the same conditions

(45) $$\sum_{l}\left\{-K\left(X_{i},X_{l}\right)\nabla U\left(X_{l}\right)+\nabla_{l}K\left(X_{i},X_{l}\right)\right\}=0,$$

for the ’equilibrium’ fixed points. See [44] for a discussion of these fixed points for different kernel functions. However, both dynamics differ for finite times *t*, where a single time step of SVGD is computationally simpler, being free of the matrix inversion required by our framework. The mean field limit N→∞ of Equation (44) differs from the FPE limit, and the resulting partial differential equation is nonlinear [57]. Nevertheless, it is possible to interpolate between the two particle dynamics. In fact, in the limit of a large regularisation parameter λ→∞, the inverse matrix in Equation (22) becomes diagonal, i.e., ${\left(K+\lambda I\right)}^{-1}≃\frac{1}{\lambda }I$, and we recover SVGD (Equation (44)) by introducing a rescaled time τ≐t/λ. This result could be of practical importance when the goal is to approximate the stationary distribution, irrespective of the finite-time dynamics. The SVGD combines faster matrix operations with slower relaxation times to equilibrium compared to the FPE dynamics. It would be interesting to see if an optimal computational speed of a particle algorithm might be achieved at some intermediate regularisation parameter λ.

### 7.3. Relation to Geometric Formulation of FPE Flow

Following Otto [58] and Villani [59], the FPE for the equilibrium case can be viewed as a gradient flow on the manifold of probability densities with respect to the Wasserstein metric. This formulation can be used to define an implicit Euler time discretisation method for the dynamics of the density $p_{t}$. For small times δt (and $\sigma^{2}=2$) this is given by the variational problem

(46) $$p_{t+\delta t}=\arg\underset{p}{\inf}\left(W_{2}^{2}\left(p,p_{t}\right)+\delta tD\left(p∥p_{\infty }\right)\right)$$

in terms of the Kullback–Leibler divergence and the $L_{2}$*Wasserstein distance*$W_{2}$. The latter gives the minimum of $\langle ∥X-X\left(t\right){∥}^{2}\rangle$ for two random variables X(t) and *X*, where the expectation is over the joint distribution with fixed marginals $p_{t}$ and *p*. Using the dual formulation for a regularised Wasserstein distance, approximate numerical algorithms for solving Equation (46) have been developed by [60] and by [61] with applications to simulations of FPE.

We show in the following that Equation (46) may be cast into a form closely related to our variational formulation (Equation (7)) for r(x)=−∇U(x). Assuming that *X* and X(t) are related through deterministic (transport) mappings of the form

(47) X=X(t)+δt∇ψ(X(t)),

we may represent the Wasserstein distance in terms of ψ and the variational problem in Equation (46) may be reformulated as

(48) $$\psi^{*}=\arg\min_{\nabla \psi }\frac{\delta t^{2}}{2}\int {∥\nabla \psi \left(x\right)∥}^{2}p_{t}\left(x\right)dx+\delta tD\left(p_{t+dt}∥p_{\infty }\right).$$

where

(49) $$p_{t+\delta t}\left(x\right)=p_{t}\left(x\right)-\delta t\nabla \cdot \left(p_{t}\left(x\right)\nabla \psi \left(x\right)\right)+O(\delta t^{2}).$$

To proceed, we expand the relative entropy in Equation (48) to first order in δt, inserting the representation of Equation (49) for $p_{t+\delta t}\left(x\right)$, thereby obtaining

(50a) $$\begin{array}{cc} \; & \frac{\delta t}{2}\int {∥\nabla \psi \left(x\right)∥}^{2}p_{t}\left(x\right)dx+D\left(p_{t+\delta t}∥p_{\infty }\right)=D\left(p_{t}∥p_{\infty }\right)+ \end{array}$$

(50b) $$\begin{array}{cc} \; & +\frac{\delta t}{2}\left(\int p_{t}\left(x\right)\left\{{∥\nabla \psi \left(x\right)∥}^{2}-2\nabla^{2}\psi \left(x\right)+2\nabla U\left(x\right)\cdot \nabla \psi \left(x\right)\right\}dx\right)+O\left(\delta t^{2}\right). \end{array}$$

Minimisation ignoring the $O(\delta t^{2})$ terms (employing integration by parts) yields

(51) $$\nabla \psi^{*}\left(x\right)=-\nabla U\left(x\right)-\nabla \ln p_{t}\left(x\right),$$

which is related to our cost function (Equation (8)) if we formally identify $\phi \left(x\right)=-\partial_{\alpha }\psi \left(x\right)$. More precisely, by replacing $p_{t}$ by samples, the empirical cost function may be regularised with a RKHS norm penalty resulting in a nonparametric estimator for unnormalised log–density $\psi^{*}\left(x\right)=-\ln p_{t}\left(x\right)-U\left(x\right)+const$, as shown in [62]. One could use this estimator as an alternative to our approach. This would lead to a simultaneous estimate of all components of the GLD. In our approach, each of the *d* components of the gradient is computed individually. In this way, we avoid additional second derivatives of kernels, which would increase the dimensionality of the resulting matrices.

## 8. Extension to General Diffusion Processes

The Fokker–Planck equations for an SDE with arbitrary drift f(x) and general, state-dependent diffusion matrix D(x) is given by

(52) $$\frac{\partial p_{t}\left(x\right)}{\partial t}=\nabla \cdot \left[-f\left(x\right)p_{t}\left(x\right)+\frac{1}{2}\nabla \cdot \left(D\left(x\right)\;p_{t}\left(x\right)\right)\right].$$

This may again be written in the form of a Liouville equation (Equation (3)) where the effective force term equals

(53) $$g\left(x,t\right)=f\left(x\right)-\frac{1}{2}\nabla \cdot D\left(x\right)-\frac{1}{2}D\left(x\right)\nabla \ln p_{t}\left(x\right).$$

## 9. Second Order Langevin Dynamics (Kramer’s Equation)

For second-order Langevin equations, the system state comprises positions $X\in R^{d}$ and velocities $V\in R^{d}$ following the coupled SDE

(54) $$\begin{array}{cc} dX & =Vdt \end{array}$$

(55) $$\begin{array}{cc} dV & =\left(-\gamma V+f(X)\right)dt+\sigma dB_{t}. \end{array}$$

In Equation (54), the dynamics describe the effect of a friction force, γV, an external force, f(X), and a fluctuating force, where γ denotes the dissipation constant. In this setting, the effective *deterministic* ODE system is given by

(56) $$\begin{array}{cc} \frac{dX}{dt} & =V\\ \frac{dV}{dt} & =-\gamma V+f\left(X\right)-\frac{\sigma^{2}}{2}\nabla_{v}\ln p_{t}\left(X,V\right). \end{array}$$

Considering here the equilibrium case, we set f(x)=−∇U(x) for which the stationary density equals

(57) $$\ln p_{\infty }\left(X,V\right)=-\beta \left(\frac{{∥V∥}^{2}}{2}+U\left(X\right)\right)\equiv -\beta H\left(X,V\right),$$

where $\beta =\frac{2\gamma }{\sigma^{2}}$ and $H\left(x,v\right)=\frac{{∥V∥}^{2}}{2}+U\left(x\right)$ denotes the *Hamiltonian* function. Inserting $p_{\infty }$ into Equation (56), we find that for t→∞, the damping and the density-dependent part of the force cancel and we are left with pure Hamiltonian dynamics

(58) $$\begin{array}{cc} \frac{dX}{dt} & =V\\ \frac{dV}{dt} & =-\nabla U(X), \end{array}$$

for which all particles become completely *decoupled*, with each one conserving energy separately. Of course, this result also precludes fixed point solutions to the particle dynamics. However, this limiting dynamics captured by Equation (58) assumes the mean field limit N→∞ together with a consistent estimate of the GLD before taking the limit t→∞. For GLD estimators at finite *N*, we expect reasonably small stationary fluctuations of individual particle energies, which were also evident in our numerical experiments.

The exact asymptotic behaviour is also reflected in the expression for the change of the relative entropy for Kramer’s equation. Similar to Equation (42) we obtain

(59) $$\begin{array}{cc} \frac{d}{dt}D\left(p_{t}|p_{\infty }\right) & =-\frac{\sigma^{2}}{2}\int p_{t}\left(x,v\right){∥\nabla_{v}\ln p_{t}\left(x,v\right)-\nabla_{v}\ln p_{\infty }\left(x,v\right)∥}^{2}dxdv\\ & =-\frac{2}{\sigma^{2}}\int p_{t}\left(x,v\right){∥\gamma v+\frac{\sigma^{2}}{2}\nabla_{v}\ln p\left(x,v\right)∥}^{2}dxdv. \end{array}$$

When the system approaches equilibrium, both terms in the norm cancel out and the entropy production rate converges to 0.

## 10. Simulating Accurate Fokker–Planck Solutions for Model Systems

To demonstrate the accuracy of our approach, we simulated solutions of FPEs for a range of model systems and compared the results with those obtained from direct stochastic simulations (Monte Carlo sampling) with the same particle number, as well as with analytic solutions where relevant. We tested our framework on systems with diverse degrees of nonlinearity and dimensionality, as well as with various types of noise (additive/multiplicative). We quantified the accuracy of transient and steady state solutions resulting from our method in terms of 1-Wasserstein distance [59] and Kullback–Leibler (KL) divergence (Appendix C and Appendix D), along with the squared error of distances between distribution cumulants. For evaluating particle solutions for nonlinear processes, where analytical solutions of the Fokker–Planck equation are intractable, we simulated a very large number ($N^{\infty }$) of stochastic trajectories that we considered to be ground truth Fokker–Planck solutions. We employed an Euler–Maruyama and forward Euler integration scheme of constant step size $dt=10^{-3}$ for stochastic and deterministic simulations respectively. We provide a description of the employed algorithm along with analysis of its computational complexity in Appendix H, while further numerical experiments on the influence of hyperparameter values on the performance of the estimator are provided in Appendix G and Appendix F.

### 10.1. Linear Conservative System with Additive Noise

**Figure 1:**
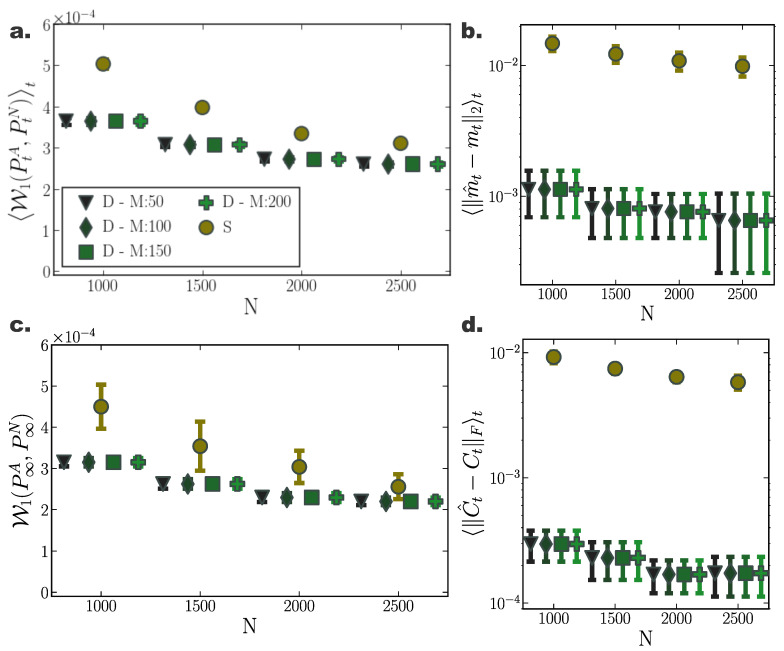
**Accuracy of Fokker–Planck solutions for two dimensional Ornstein Uhlenbeck process.** (**a**) Mean, ${\langle W_{1}\left(P_{t}^{A},P_{t}^{N}\right)\rangle }_{t}$, and (**c**) stationary $W_{1}\left(P_{\infty }^{A},P_{\infty }^{N}\right)$, 1-Wasserstein distance, between analytic solution and deterministic(D)/stochastic(S) simulations of *N* particles (for different inducing point number *M*). (**b**) Average temporal deviations from analytic mean $m_{t}$ and (**d**) covariance matrix $C_{t}$ for deterministic and stochastic system for increasing particle number *N*. Deterministic particle simulations consistently outperformed stochastic ones in approximating the temporal evolution of the mean and covariance of the distribution for all examined particle number settings. (Further parameter values: regularisation constant λ=0.001, Euler integration time step $dt=10^{-3}$, and RBF kernel length scale *l* estimated at every time point as two times the standard deviation of the state vector. Inducing point locations were selected randomly at each time step from a uniform distribution spanning the state space volume covered by the state vector).

For a two dimensional Ornstein-Uhlenbeck process (Appendix A.1) transient and stationary densities evolved through deterministic particle simulations (D) consistently outperformed their stochastic counterparts (S) comprising the same number of particles in terms accuracy in approximating the underlying density (Figure 1). In particular, comparing the 1-Wasserstein distance between samples from analytically derived densities ($P_{t}^{A}$) (Appendix B)—considered here to reflect the ground truth—and the deterministically (D) or stochastically (S) evolved densities ($P_{t}^{N}$), $W_{1}\left(P_{t}^{A},P_{t}^{N}\right)$, we observed smaller Wasserstein distances to ground truth for densities evolved according to our deterministic particle dynamics, both for transient (Figure 1a) and stationary (Figure 1c) solutions. Specifically, we quantified the transient deviation of simulated densities from ground truth by the average temporal 1-Wasserstein distance, ${\langle W_{1}\left(P_{t}^{A},P_{t}^{N}\right)\rangle }_{t}$ (Appendix D). For small particle number, deterministically evolved interacting particle trajectories represented more reliably the evolution of the true probability density compared to independent stochastic ones, as portrayed by smaller average Wasserstein distances. For increasing particle number, the accuracy of the simulated solutions with the two approaches converged. Yet, while for N=2500 particles the stochastically evolved densities suggest *on average* (over trials) comparable approximation precision with their deterministic counterparts, the deterministically evolved densities more reliably delivered densities of a certain accuracy, as proclaimed by the smaller dispersion of Wasserstein distances among different realisations (Figure 1a,c).

Likewise, we observed similar results when comparing only the stationary distributions, $W_{1}\left(P_{\infty }^{A},P_{\infty }^{N}\right)$ (Figure 1c). While for small particle number, the interacting particle system more accurately captured the underlying limiting distribution, for increasing particle number the accuracy of both approaches converged, with our method consistently delivering more reliable approximations among individual repetitions.

**Figure 2:**
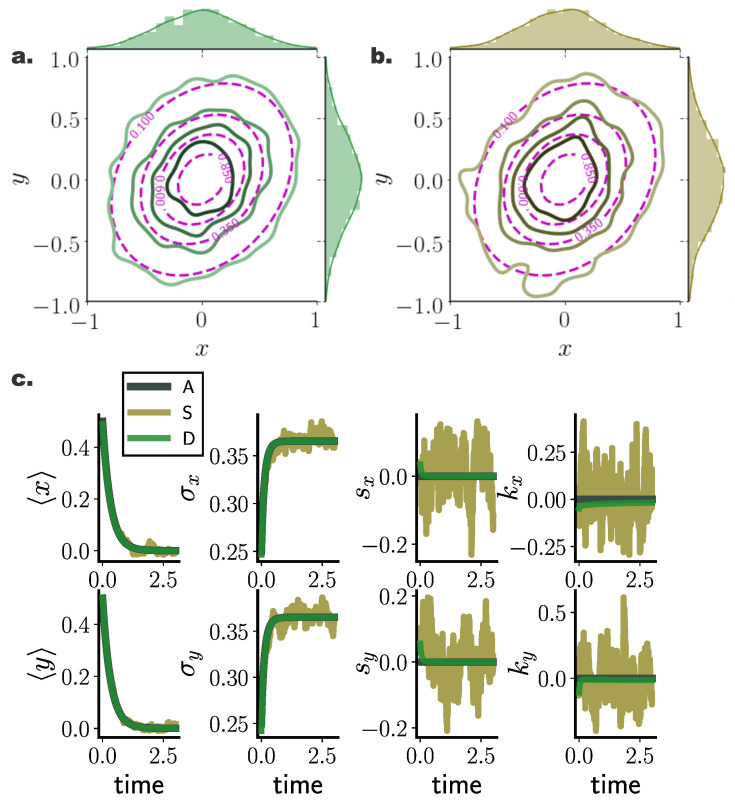
**Stationary and transient Fokker–Planck solutions computed with deterministic (green) and stochastic (brown) particle dynamics for a two dimensional Ornstein Uhlenbeck process.** (**a**,**b**) Estimated stationary PDFs arising from deterministic (N=1000) (green), and stochastic (N=1000) (brown) particle dynamics. Purple contours denote analytically calculated stationary distributions, while top and side histograms display marginal distributions for each dimension. (**c**) Temporal evolution of marginal statistics, mean 〈x〉, standard deviation $\sigma_{x}$, skewness $s_{x}$, and kurtosis $k_{x}$, for analytic solution (*A*), and for stochastic (*S*) and deterministic (*D*) particle systems comprising N=1000, with initial state distribution $N\left(\left[\begin{array}{c} 0.5\\ 0.5 \end{array}\right],\left[\begin{array}{cc} 0.05^{2} & 0\\ 0 & 0.05^{2} \end{array}\right]\right)$, for M=100 randomly selected inducing points employed in the gradient–log–density estimation. Deterministic particle simulations deliver smooth cumulant trajectories, as opposed to highly fluctuating stochastic particle cumulants. (Further parameter values: regularisation constant λ=0.001, and RBF kernel length scale *l* estimated at every time point as two times the standard deviation of the state vector. Inducing point locations were selected randomly at each time step from a uniform distribution spanning the state space volume covered by the state vector).

Moreover, densities evolved with our deterministic framework exhibited less fluctuating cumulant trajectories in time, compared to their stochastic counterparts (Figure 2c). In particular, even for limited particle number, cumulants calculated over deterministically evolved particles progressed smoothly in time, while substantially more particles for the stochastic simulations were required for the same temporal cumulant smoothness. To further quantify the transient accuracy of Fokker–Planck solutions computed with our method, we compared the average transient discrepancy between the first two analytic cumulants ($m_{t}$ and $C_{t}$) to those estimated from the particles (${\hat{m}}_{t}$ and ${\hat{C}}_{t}$), $\langle ∥{\hat{m}}_{t}-m_{t}{{∥}_{2}\rangle }_{t}$ (Figure 1b) and $\langle ∥{\hat{C}}_{t}-C_{t}{{∥}_{F}\rangle }_{t}$ (Figure 1d), where ${∥\cdot ∥}_{F}$ stands for the Frobenious norm (Appendix E). In line with our previous results, our deterministic framework delivered considerably more accurate transient cumulants when compared to stochastic simulations, with more consistent results among individual realisations, denoted by smaller dispersion of average cumulant differences. (Notice the logarithmic y-axis scale in Figure 1b,d. Error bars for the stochastic solutions were in fact larger than those for the deterministic solutions on a linear scale.)

Interestingly, the number of sparse points *M* employed in the gradient–log–density estimation had only minor influence on the quality of the solution (Figure 1a,c). This hints to substantially low computational demands for obtaining accurate Fokker–Planck solutions, since our method is computationally limited by the inversion of the M×M matrix in Equation (29).

### 10.2. Bi-Stable Nonlinear System with Additive Noise

For nonlinear processes, since the transient solution of the FPE is analytically intractable, we compared the transient and stationary densities estimated by our method with those returned from stochastic simulations of $N^{\infty }$ = 26,000 particles, and contrasted them against stochastic simulations with the same particle number.

**Figure 3:**
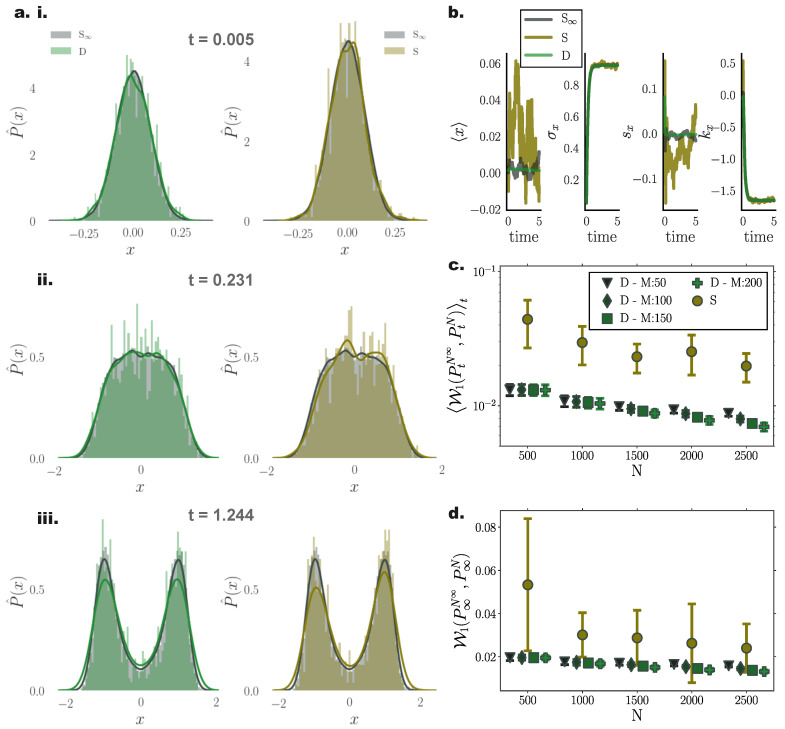
**Performance of deterministic (green) and stochastic (brown) *N* particle solutions compared to $N^{\infty }$ (grey) stochastic particle densities for a nonlinear bi-stable process.** (**a**) Instances of estimated pdfs arising from (**left**) stochastic ($N^{\infty }$ = 26,000) (grey) and deterministic (N=1000) (green), and (**right**) stochastic ($N^{\infty }=26,000$) (grey) and stochastic (N=1000) (brown) particle dynamics at times (**i**) t=0.005, (**ii**) t=0.231, and (**iii**) t=1.244. (**b**) Temporal evolution of first four distribution cumulants, mean 〈x〉, standard deviation $\sigma_{x}$, skewness $s_{x}$, and kurtosis $k_{x}$, for stochastic ($S^{\infty }$ and *S*) and deterministic (*D*) systems comprising $N^{\infty }$ = 26,000, N=1000, with initial state distribution $N(0,0.05^{2})$, by employing M=150 inducing points in the gradient–log–density estimation. (**c**) Mean, ${\langle W_{1}\left(P_{t}^{N^{\infty }},P_{t}^{N}\right)\rangle }_{t}$, and (**d**) stationary, $W_{1}\left(P_{\infty }^{A},P_{\infty }^{N}\right)$, 1-Wasserstein distance, between $N^{\infty }$ = 26,000 stochastic, and deterministic (D)/stochastic (S) simulations of *N* particles (for different inducing point number *M*). (Further parameter values: regularisation constant λ=0.001, Euler integration time step $dt=10^{-3}$, and RBF kernel length scale l=0.5. Inducing point locations were selected randomly at each time step from a uniform distribution spanning the state space volume covered by the state vector).

For a system with bi-modal stationary distribution (Appendix A.2), the resulting particle densities from our deterministic framework closely agreed with those arising from the stochastic system with $N^{\infty }$ = 26,000 particles (Figure 3a). In particular, deterministically evolved distributions respected the symmetry of the underlying double–well potential, while the stochastic system failed to accurately capture the potential symmetric structure Figure 3a (iii).

Systematic comparisons of the 1-Wasserstein distance between deterministic and stochastic *N* particle simulations with the “$N^{\infty }$” stochastic simulation comprising $N^{\infty }=26,000$ particles revealed that our approach efficiently captured the underlying PDF already with N=500 particles (Figure 3c,d). For increasing particle number, the stationary solutions of both systems converged to the “$N^{\infty }$” one. However, we observed a systematically increasing approximation accuracy delivered from the deterministic simulations compared to their stochastic counterparts.

It is noteworthy that, on average, deterministic simulations of N=500 particles conveyed a better approximation of the underlying transient PDF compared to stochastic simulations of N=2500 particles (Figure 3c).

Interestingly, for small particle number, the number of employed inducing points *M* did not significantly influence the accuracy of the approximated solution. However for increasing particle number, enlarging the set of inducing points contributed to more accurate approximation of Fokker–Planck equation solutions (Figure 3c), with the trade off of additional computational cost.

Similar to the Ornstein Uhlenbeck process (Section 10.1), comparing cumulant trajectories computed from both the deterministic and stochastic particle systems revealed less fluctuating cumulant evolution for densities evolved with our deterministic framework also in this nonlinear setting (Figure 3b).

### 10.3. Nonlinear System Perturbed by Multiplicative Noise

**Figure 4:**
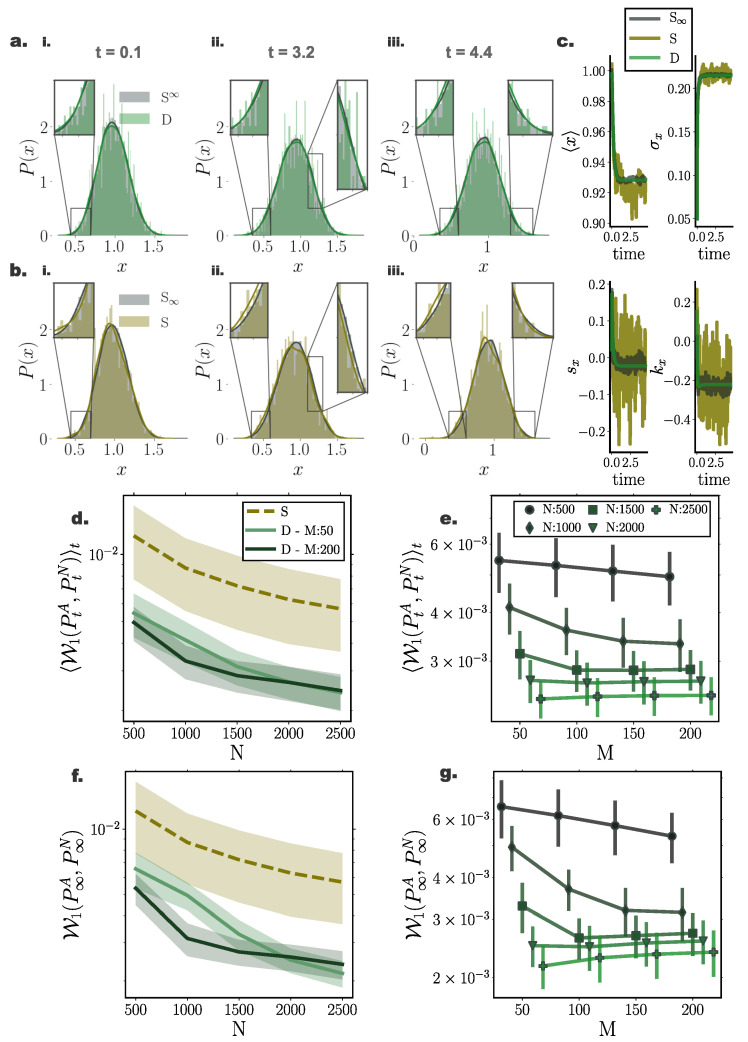
**Accuracy of Fokker–Planck solutions for a nonlinear system perturbed with state-dependent noise.** (**a**) Instances of N=1000 particle distributions resulting from deterministic (green) and (**b**) stochastic (brown) simulations against stochastic particle distributions comprising $N^{\infty }$ = 35,000 particles (grey) for (**i**) t=0.1, (**ii**) t=3.2, and (**iii**) t=4.4. Insets provide a closer view of details of distribution for visual clarity. Distributions resulting from deterministic particle simulations closer agree with underlying distribution for all three instances. (**c**) Temporal evolution of first four cumulants for the three particle systems (grey: $S_{\infty }$—stochastic with $N^{\infty }$ = 35000 particles, brown: *S* - stochastic with *N* = 1000 particles, and green: *D*—deterministic with *N* = 1000 particles). Deterministically evolved distributions result in smooth cumulant trajectories. (**d**,**e**) Temporal average and (**f**,**g**) stationary 1-Wasserstein distance between distributions mediated through stochastic simulations of $N^{\infty }$ = 35000, and through deterministic (green) and stochastic (brown) simulations of *N* particles against particle number *N* and inducing point number *M*. Shaded regions and error bars denote one standard deviation among 20 independent repetitions. Different green hues designate different inducing point number *M* employed in the gradient–log–density estimation. (Further parameter values: regularisation constant λ=0.001, Euler integration time step $dt=10^{-3}$, and RBF kernel length scale l=0.25. Inducing points were arranged on a regular grid spanning the instantaneous state space volume captured by the state vector).

To assess the accuracy of our framework on general diffusion processes perturbed by state-dependent (multiplicative) noise, we simulated a bi-stable system with dynamics governed by Equation (A3) with diffusion function $D\left(x\right)=sin^{2}\left(x\right)$ according to Equation (53). Similarly, in this setting, deterministic particle distributions delivered a closer approximation of the underlying density when compared to direct stochastic simulations. In particular, we found that in this setting, deterministically evolved distributions more accurately captured the tails of the underlying distribution, mediated here by stochastic simulations of $N^{\infty }$ = 35,000 particles (Figure 4a,b).

Similar to the previously examined settings, the deterministic framework delivered more reliable and smooth trajectories for the marginal statistics of the underlying distribution (Figure 4c).

Comparing the temporal average and stationary 1-Wasserstein distance (Figure 4d,f) between the optimal stochastic distributions and the deterministic and stochastic particle distributions of size *N*, we found that the deterministic system delivered consistently more accurate approximations, as portrayed by smaller 1-Wasserstein distances.

Interestingly, we found that for deterministic particle simulations, the number of employed sparse points in the gradient–log–density estimation mediated a moderate approximation improvement for small system sizes, while for systems comprising more than N=2000 particles, the number of sparse points had minimal or no influence on the accuracy of the resulting distribution (Figure 4e,g).

### 10.4. Performance in Higher Dimensions

**Figure 5:**
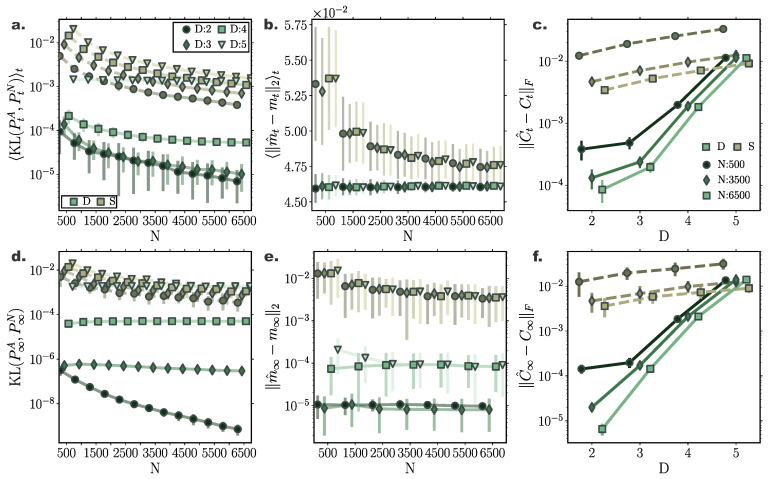
**Accuracy of Fokker-Planck solutions for multi-dimensional Ornstein–Uhlenbeck processes for M=100 inducing points.** Comparison of deterministic particle Fokker–Planck solutions with stochastic particle systems and analytic solutions for multi-dimensional Ornstein–Uhlenbeck process of D = {2, 3, 4, 5} dimensions. (**a**) Time-averaged and (**d**) stationary Kullback–Leibler (KL) divergence between simulated particle solutions (green: deterministic, brown: stochastic) and analytic solutions for different dimensions. Deterministic particle simulations outperform stochastic particle solutions even for increasing system dimensionality. (**b**) Time averaged and (**e**) stationary error between analytic, $m_{t}$, and sample mean, ${\hat{m}}_{t}$, for increasing particle number. (**c**) Time averaged and (**f**) stationary discrepancy between simulated, ${\hat{C}}_{t}$, and analytic covariances, $C_{t}$, as captured by the Frobenius norm of the relevant covariance matrices difference. The accuracy of the estimated covariance decreases for increasing dimensionality. (Further parameter values: regularisation constant λ=0.001, Euler integration time step $dt=10^{-3}$, and adaptive RBF kernel length scale *l* calculated at every time step as two times the standard deviation of the state vector. Inducing point locations were selected randomly at each time step from a uniform distribution spanning the state space volume covered by the state vector).

**Figure 6:**
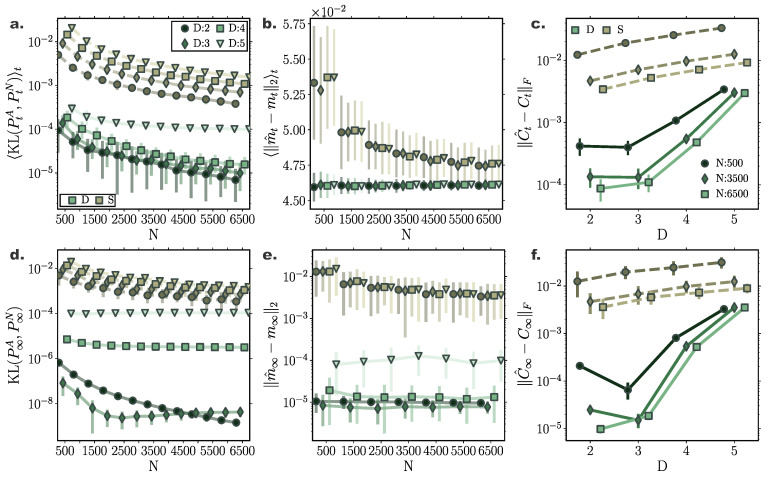
**Accuracy of Fokker-Planck solutions for multi-dimensional Ornstein–Uhlenbeck processes for M=200 inducing points.** Comparison of deterministic particle Fokker–Planck solutions with stochastic particle systems and analytic solutions for multi-dimensional Ornstein–Uhlenbeck process of D = {2,3,4,5} dimensions. (**a**) Time-averaged and (**d**) stationary Kullback–Leibler (KL) divergence between simulated particle solutions (green: deterministic, brown: stochastic) and analytic solutions for different dimensions. Deterministic particle simulations outperform stochastic particle solutions even for increasing system dimensionality. (**b**) Time averaged and (**e**) stationary error between analytic, $m_{t}$, and sample mean, ${\hat{m}}_{t}$, for increasing particle number. (**c**) Time averaged and (**f**) stationary discrepancy between simulated, ${\hat{C}}_{t}$, and analytic covariances, $C_{t}$, as captured by the Frobenius norm of the relevant covariance matrices difference. The accuracy of the estimated covariance decreases for increasing dimensionality. (Further parameter values: regularisation constant λ=0.001, Euler integration time step $dt=10^{-3}$, and adaptive RBF kernel length scale *l* calculated at every time step as two times the standard deviation of the state vector. Inducing point locations were selected randomly at each time step from a uniform distribution spanning the state space volume covered by the state vector).

To quantify the scaling and performance of the proposed framework for increasing system dimension, we systematically compared simulated densities with analytically calculated ones for Ornstein–Uhlenbeck processes of dimension D={2,3,4,5} following the dynamics of Equation (A4) for inducing point number M=100 (Figure 5) and M=200 (Figure 6). To evaluate simulated Fokker–Planck solutions, we calculated the Kullback–Leibler divergence between analytically evolved densities (Appendix B) and particle densities. We employed the closed-form equation for estimating KL divergence between two Gaussian distributions (Appendix C) for empirically estimated mean, ${\hat{m}}_{t}$, and covariance, ${\hat{C}}_{t}$, for particle distributions.

For all dimensionalities, the deterministic particle solutions approximated transient and stationary densities remarkably accurately with Kullback–Leibler divergence between the simulated and analytically derived densities below $10^{-2}$ for all dimensions, both for transient and stationary solutions (Figure 5a,d and Figure 6a,d). In fact, the deterministic particle solutions delivered more precise approximations of the underlying densities compared to direct stochastic simulations of the same particle number. Remarkably, even for processes of dimension D=5, deterministically evolved solutions mediated through N=500 particles resulted in approximately the same KL divergence of stochastic particle solutions of N=6500 particles.

Our deterministic particle method delivered consistently better approximations of the mean of the underlying densities compared to stochastic particle simulations (Figure 5b,e). Specifically, estimations of the stationary mean of the underlying distributions were more than two orders of magnitude accurate that their stochastically approximated counterparts already for small particle number (Figure 5e).

Yet, the accuracy of our deterministic framework deteriorated for increasing dimension (Figure 5a,d). More precisely, while for low dimensionalities the covariance matrices of the underlying densities were accurately captured by deterministically evolved particles, for increasing system dimension approximations of covariance matrices became progressively worse. Yet, even for systems of dimension D=5, covariance matrices computed from deterministically simulated solutions of N=500 particles were at the same order of magnitude as accurate as covariances delivered by stochastic particle simulations of size N=6500.

However, comparing the resulting performance of solutions delivered by employing different number of inducing points (Figure 5 and Figure 6) reveals that for increasing dimension more inducing points are required to attain accurate FPE solutions. In particular, both transient and stationary KL divergences to ground truth improved remarkably for dimensions D=4 and D=5 by employing M=200 inducing points in the gradient–log–density estimation (Figure 5a,d and Figure 6a,d). In more detail, for nearly all dimensions, the estimation of the covariance of the underlying distribution improved considerably, both for transients and stationary solutions (Figure 5c,f and Figure 6c,f), while only the stationary mean of dimension D=4 showed significant improvement (Figure 5b,e and Figure 6b,e). Figure 6c,f reveals that by increasing the number of inducing points our framework is able to capture more effectively the spread of the underlying distribution, clearly surpassing in approximation accuracy solutions mediated by stochastic particle simulations.

### 10.5. Second order Langevin Systems

**Figure 7:**
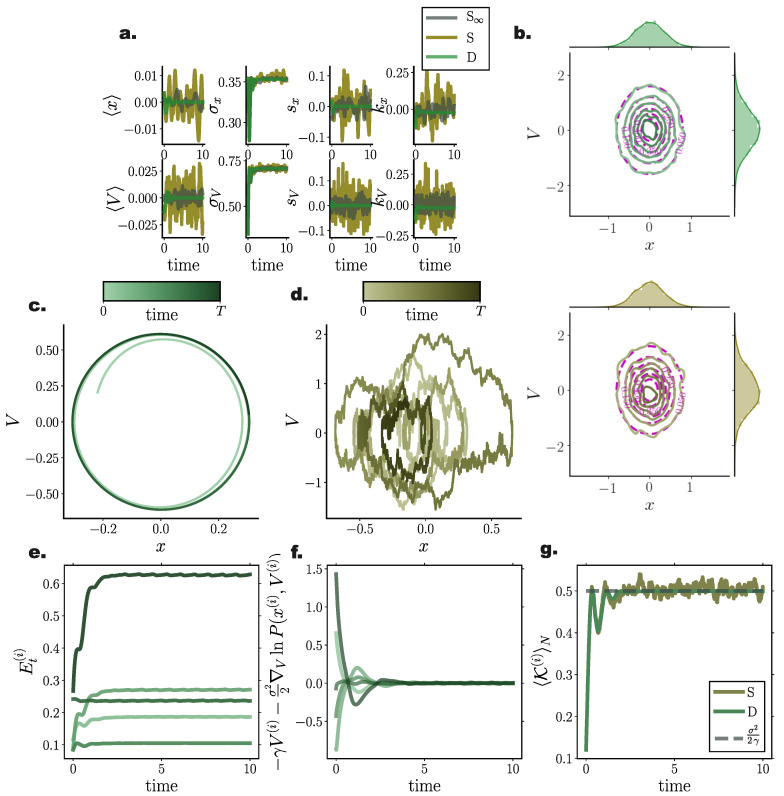
**Energy preservation for second order Langevin dynamics in a quadratic potential.** Comparison of deterministic particle Fokker–Planck solutions with stochastic particle systems for a harmonic oscillator (**a**) First four cumulant temporal evolution for deterministic (green) and stochastic (brown) system. (**b**) Stationary joint and marginal distributions for deterministic (green) and stochastic (brown) systems. Purple lines denote analytically derived stationary distributions. (**c,d**) State space trajectory of a single particle for deterministic (green) and stochastic (brown) system. Color gradients denote time. (**e**) Temporal evolution of individual particle energy $E_{t}^{\left(i\right)}$ for deterministic system for 5 particles. (**f**) Difference between velocity and gradient–log–density term for individual particles. After the system reaches stationary state the particle velocity and GLD term cancel out. (**g**) Ensemble average kinetic energy through time resorts to $\frac{\sigma^{2}}{2\;\gamma }$ (grey dashed line) after equilibrium is reached. (Further parameter values: regularisation constant λ=0.001, integration time step $dt=2\times 10^{-3}$, and adaptive RBF kernel length scale *l* calculated at every time step as two times the standard deviation of the state vector. Number of inducing points M=300. Inducing point locations were selected randomly at each time step from a uniform distribution spanning the state space volume covered by the state vector).

**Figure 8:**
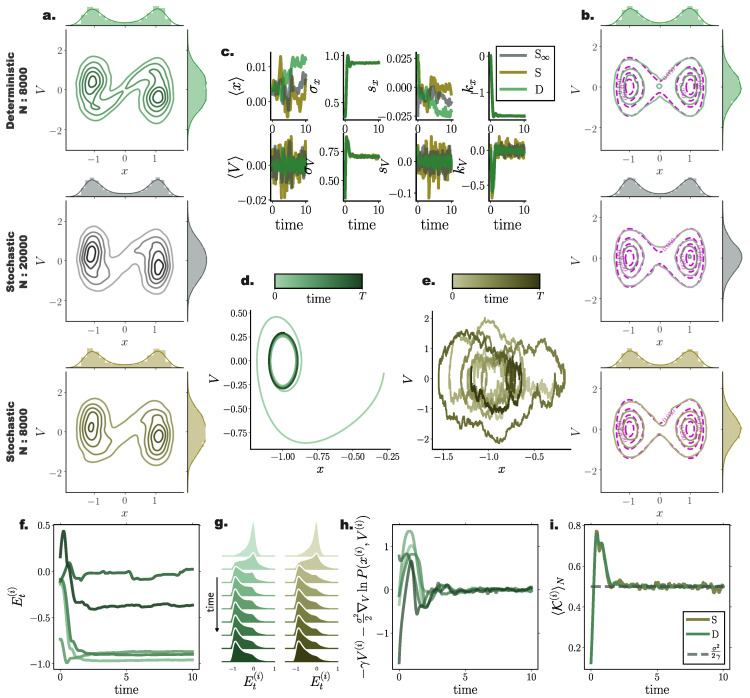
**Energy preservation for second order Langevin dynamics in a double well potential.** Comparison of deterministic particle Fokker–Planck solutions with stochastic particle systems for a bistable process. (**a**,**b**) Joint and marginal distributions of system states mediated by N=8000 particles evolved with our framework (green) and with direct stochastic simulations comprising $N^{\infty }$ = 20,000 (grey) and N=8000 (brown) particles at (**a**) t=0.6, and (**b**) t=10. Purple lines denote the analytically derived stationary density. (**c**) First four cumulant temporal evolution for deterministic (green) and stochastic (brown) system. (**d**) State space trajectory of a single particle for deterministic and (**e**) stochastic system. Color gradients denote time. (**f**) Temporal evolution of individual particle energy $E_{t}^{\left(i\right)}$ for deterministic system for 5 particles. (**g**) Temporal evolution of distribution of particle energies $E_{t}^{\left(i\right)}$ for deterministic (green) and stochastic (brown) system. (**h**) Difference between velocity and gradient log density term for individual particles. (**i**) Ensemble average kinetic energy through time resorts to $\frac{\sigma^{2}}{2\;\gamma }$ (grey dashed line) after equilibrium is reached. (Further parameter values: regularisation constant λ=0.001, integration time step $dt=2\;\times \;10^{-3}$ and adaptive RBF kernel length scale *l* calculated at every time step as two times the standard deviation of the state vector. Number of inducing points M=300. Inducing point locations were selected randomly at each time step from a uniform distribution spanning the state space volume covered by the state vector).

To demonstrate the performance of our framework for simulating solutions of the FPEs for second order Langevin systems as described in Section 9, we incorporated our method in a symplectic Verlet integrator (Equations (A10)–(A12)) simulating the second-order dynamics captured by Equation (56) for a linear f(x)=−4x and a nonlinear, $f\left(x\right)=-4\;x^{3}+4\;x$, drift function (Equation (A10)), and compared the results with stochastic simulations integrated by a semi-symplectic framework [63]. In agreement with previous results, cumulant trajectories evolved smoother in time for deterministic particle simulations when compared to their stochastic counterparts (Figure 7a and Figure 8c). Stationary densities closely matched analytically derived ones (see Equation (A7)) (purple contour lines in Figure 7b and Figure 8b), while transient densities captured the fine details of simulated stochastic particle densities comprising $N^{\infty }=20,000$ (Figure 8a).

Furthermore, the symplectic integration contributed to the preservation of energy levels for each particle after the system reached equilibrium (Figure 7e and Figure 8f), which was also evident when observing individual particle trajectories in the state space (Figure 7c,d and Figure 8d,e).

As already conveyed in Section 9, the velocity term and the gradient–log–density term canceled out in the long time limit (Figure 7f and Figure 8g) for each particle individually, while the average kinetic energy in equilibrium exactly resorted to the value dictated by the fluctuation–dissipation relation and the equipartition of energy property, i.e., ${\langle K^{\left(i\right)}\rangle }_{N}=\frac{\sigma^{2}}{2\;\gamma }$ (Figure 7g and Figure 8h).

### 10.6. Nonconservative Chaotic System with Additive Noise (Lorenz Attractor)

**Figure 9:**
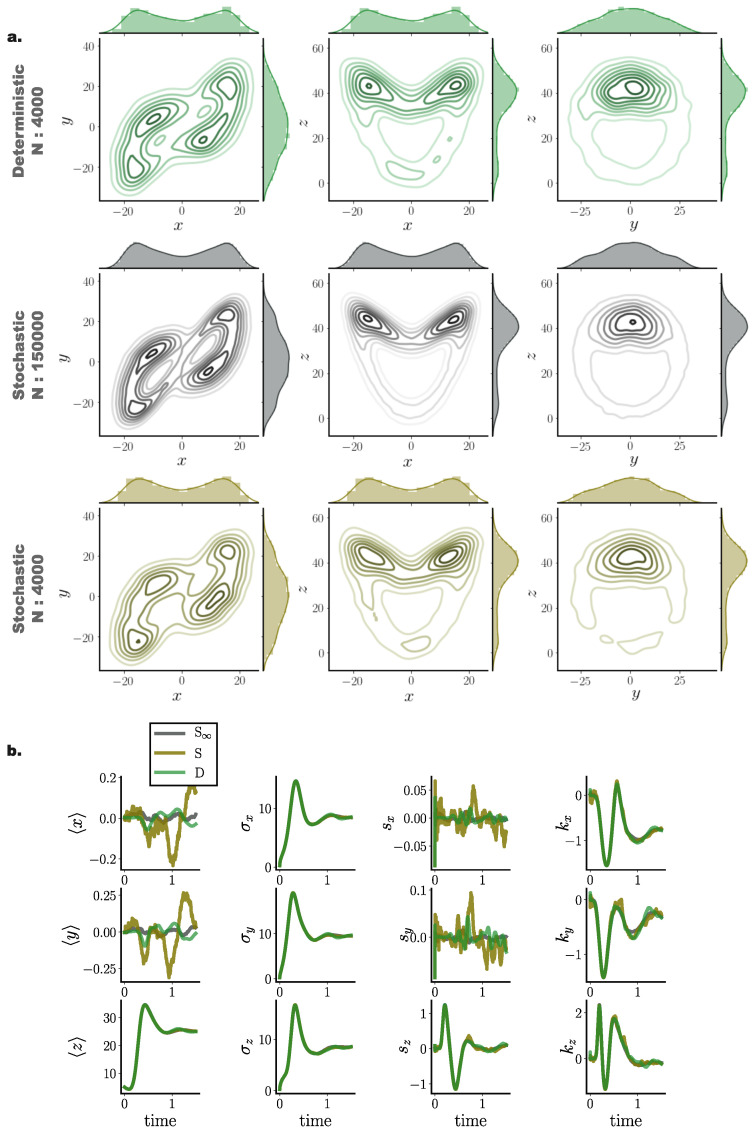
**Deterministic (green) and stochastic (brown) Fokker–Planck particle solutions for a three dimensional Lorenz attractor system in the chaotic regime perturbed by additive Gaussian noise.** (**a**) Joint and marginal distributions of system states mediated by N=4000 particles evolved with our framework (green) and with direct stochastic simulations comprising *N* = 150,000 (grey) and N=4000 (brown) particles at t=0.4. (**b**) Cumulant trajectories for the three particle systems. Cumulants derived from deterministic particle simulations (green) closer match cumulant evolution of the underlying distribution (grey) compared to stochastic simulations (brown). (Further parameter values: regularisation constant λ=0.001, Euler integration time step $dt=10^{-3}$, adaptive RBF kernel length scale *l* calculated at every time step as two times the standard deviation of the state vector. Number of inducing points: M=200. Inducing point locations were selected randomly at each time step from a uniform distribution spanning the state space volume covered by the state vector).

As a final assessment of our framework for simulating accurate solutions of Fokker–Planck equations, we employed a Lorenz attractor model with parameters rendering the dynamics chaotic, perturbed by moderate additive Gaussian noise (Equation (A13)). By comparing stochastic simulations of $N^{\infty }$ = 150,000 particles and deterministic and stochastic simulations of N=4000 particles (Figure 9), we observed that the deterministic framework more precisely captured finer details of the underlying distribution (Figure 9a), represented here by the $N^{\infty }$ stochastic simulation. While both stochastic and deterministic simulations capture the overall butterfly profile of the Lorenz attractor, the deterministic system indeed delivered a closer match to the underlying distribution.

Similar to the previously examined models, cumulant trajectories computed from deterministically evolved particles show closer agreement with those computed from the $N^{\infty }$ stochastic system, compared to the stochastic system comprising *N* particles (Figure 9b). In particular, cumulants for the *x* and *y* states exhibited high temporal fluctuations when computed from stochastically evolved distributions, while our framework conveyed more accurate cumulant trajectories, closer to those delivered by the $N^{\infty }$ stochastic system.

## 11. Discussion and Outlook

We presented a particle method for simulating solutions of FPEs governing the temporal evolution of the probability density for stochastic dynamical systems of the diffusion type. By reframing the FPE in a Liouville form, we obtained an effective dynamics in terms of independent deterministic particle trajectories. Unfortunately, this formulation requires the knowledge of the gradient of the logarithm of the instantaneous probability density of the system state, which is actually the unknown quantity of interest. We circumvented this complication by introducing statistical estimators for the gradient–log–density based on a variational formulation. To combine high flexibility of estimators with computational efficiency, we employed kernel-based estimation together with an additional sparse approximation. For the case of equilibrium systems, we related our framework to Stein Variational Gradient Descent, a particle-based dynamics to approximate the stationary density, and to a geometric formulation of Fokker–Planck dynamics. We further discussed extensions of our method to settings with multiplicative noise and to second order Langevin dynamics.

To demonstrate the performance of our framework, we provided detailed tests and comparisons with stochastic simulations and analytic solutions (when possible). We demonstrated the accuracy of our method on conservative and non-conservative model systems with different dimensionalities. In particular, we found that our framework outperforms stochastic simulations both in linear and nonlinear settings by delivering more accurate densities for small particle number when the dimensionality is small enough. For increasing particle number, the accuracy of both approaches converges. Yet, our deterministic framework *consistently* delivered results with smaller variance among individual repetitions. Furthermore, we showed that our method, even for small particle numbers, exhibits low-order cumulant trajectories with significantly less temporal fluctuations when compared against to stochastic simulations of the same particle number.

We envisage several ways to improve and extend our method. There is room for enhancement by optimising hyperparameters of our algorithm such as inducing point position and kernel length scale. Current grid-based and uniform random selection of inducing point position may contribute to the deterioration of solution accuracy in higher dimensions. Other methods, such as subsampling or clustering of particle positions may lead to further improvements. On the other hand, a hyperparameter update may not be at all necessary at each time step in certain settings, such that a further speedup of our algorithm could be achieved.

The implementation of our method depends on the function class chosen to represent the estimator. In this paper we have focused on linear representations, leading to simple closed form expressions. It would be interesting to see if other, nonlinear parametric models, such as neural networks, (see e.g., [64]) could be employed to represent estimators. While in this setting, there would be no closed-form solutions, the small changes in estimates between successive time steps suggest that only a few updates of numerical optimisation may be necessary at each step. Moreover, the ability of neural networks to automatically learn relevant features from data might help to improve performance for higher dimensional problems when particle motion is typically restricted on lower dimensional submanifolds.

From a theoretical point of view, rigorous results on the accuracy of the particle approximation would be important. These would depend on the speed of convergence of estimators towards exact gradients of log–densities. However, to obtain such results may not be easy. While rates of convergence for kernel-based estimators have been studied in the literature, the methods for proofs usually rely on the independence of samples and would not necessarily apply to the case of interacting particles.

We have so far addressed only the forward simulation of FPEs. However, preliminary results indicate that related techniques may be applied to particle based simulations for smoothing (forward–backward) and related control problems for diffusion processes [65]. Such problems involve computations of an effective, controlled drift function in terms of gradient–log–densities. We defer further details and discussions on subsequent publications on the topic.

Taken together, the main advantage of our framework is its minimal requirement in simulated particle trajectories for attaining reliable Fokker–Planck solutions with smoothly evolving transient statistics. Moreover, our proposed method is nearly effortless to set up when compared to classical grid-based FPE solvers, while it delivers more reliable results than direct stochastic simulations.

## Figures and Tables

**Figure A1:**
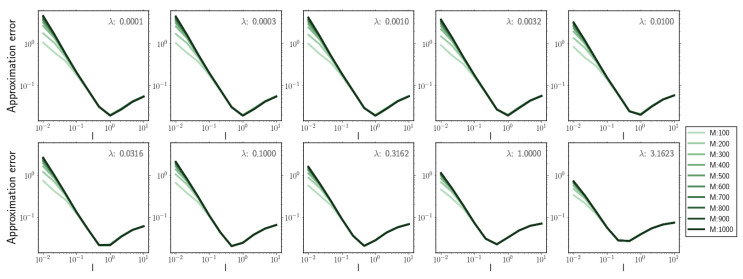
Approximation error for increasing kernel length scale *l* for different regularisation parameter values λ and inducing point number *M*.

**Figure A2:**
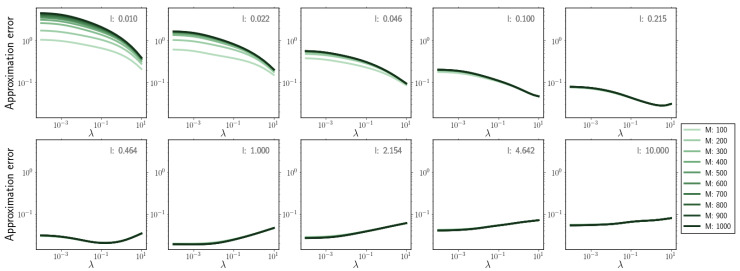
Approximation error for increasing regularisation parameter value λ for different kernel length scale *l* and inducing point number *M*.

**Figure A3:**
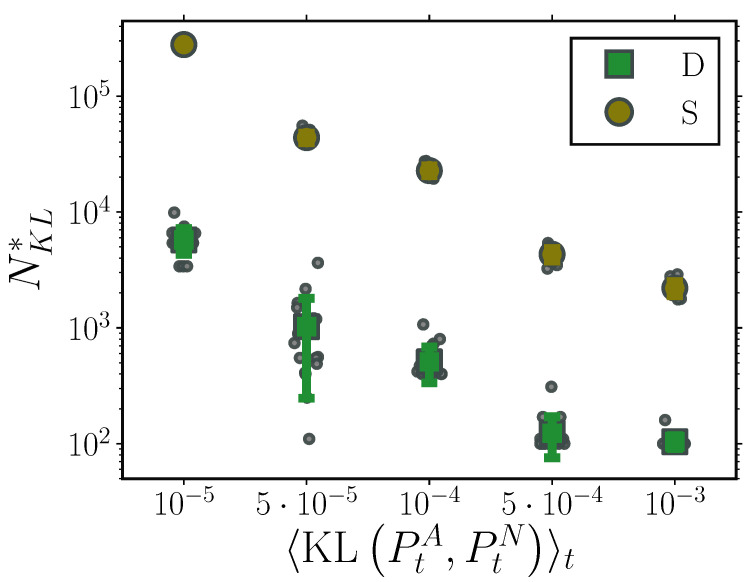
Required particle number, $N_{KL}^{*}$, to attain time averaged Kullback–Leibler divergence to ground truth, ${\langle \mathrm{KL}\left(P_{t}^{A},P_{t}^{N}\right)\rangle }_{t}$, for deterministic (green) and stochastic (brown) particle systems for a two dimensional Ornstein-Uhlenbeck process. Markers indicate mean required particle number, while error bars denote one standard deviation over 20 independent realisations. Grey circles indicate required particle number for each individual realisation. The deterministic particle system consistently required at least one order of magnitude less particles compared to its stochastic counterpart. (Further parameter values: regularisation constant λ=0.001, inducing point number M=100, and RBF kernel length scale *l* estimated at every time point as two times the standard deviation of the state vector. Inducing point locations were selected randomly at each time step from a uniform distribution spanning the state space volume covered by the state vector).

## Appendix A. Simulated Systems

### Appendix A.1. Two Dimensional Ornstein-Uhlenbeck Process

For comparing Fokker-Planck solutions computed with our approach with solutions derived from stochastic simulations, we considered the two dimensional Ornstein-Uhlenbeck process captured by the following equations

(A1) $$\begin{array}{cc} dX_{t} & =\left(-4X_{t}+Y_{t}\right)dt+\sigma \;dB_{1} \end{array}$$

(A2) $$\begin{array}{cc} dY_{t} & =\left(-4Y_{t}+X_{t}\right)dt+\sigma \;dB_{2}, \end{array}$$

where the related potential is $U\left(x,y\right)=2x^{2}-x\;y+2y^{2}$. The simulation time was set to T=3 with Euler–Maruyama integration step $dt=10^{-3}$. For estimating the instantaneous gradient log density we employed M={50,100,150,200} inducing points, randomly selected at every time point from a uniform distribution spanning the state space volume covered by the particles at the current time point.

### Appendix A.2. Bistable Nonlinear System

For testing our framework on nonlinear settings, we simulated

(A3) $$\begin{array}{cc} dX_{t} & =\left(-4{X_{t}}^{3}+4X_{t}\right)dt+\;D{\left(X_{t}\right)}^{\frac{1}{2}}dB_{t}, \end{array}$$

with D(x)=1 for evaluating solutions with additive Gaussian noise, and with $D\left(x\right)=sin^{2}\left(x\right)$ for multiplicative noise FP solutions. The associated potential is $U\left(x\right)=x^{4}-2x^{2}$.

### Appendix A.3. Multi-Dimensional Ornstein-Uhlenbeck Processes

For quantifying the scaling of our method for increasing system dimension, we simulated systems of dimensionality D={2,3,4,5} according to the following equation

(A4) $$d{X_{\left(i\right)}}_{t}=\left(-4{X_{\left(i\right)}}_{t}+\sum_{j=1,i\neq j}^{d}\frac{1}{2}{X_{\left(j\right)}}_{t}\right)dt+dB_{\left(i\right)},$$

for d∈D. Simulation time was determined by the time required for analytic mean $m_{t}$ to converge to its stationary solution within $\tilde{\epsilon }$ precision $\tilde{\epsilon }=10^{-5}$, while the integration step was set to $dt=10^{-3}$.

### Appendix A.4. Second Order Langevin Dynamics

For demonstrating the energy preservation properties of our method for second-order Langevin dynamics, we incorporated our framework into a Verlet symplectic integration scheme (Equation (A10)), and compared the results with stochastic simulations integrated according to a semi-symplectic scheme [63].

We consider a system with dynamics for positions *X* and velocities *V* captured by

(A5) $$\begin{array}{cc} dX & =Vdt \end{array}$$

(A6) $$\begin{array}{cc} dV & =\left(-\gamma V+f(X)\right)dt+\sigma dB_{t}\;, \end{array}$$

where the velocity change (acceleration) is the sum of a deterministic drift *f*, a velocity-dependent damping −γV, and a stochastic noise term $\sigma dB_{t}$.

In conservative settings, the drift is the gradient of a potential f(x)=−∇U(x). Here we used a quadratic (harmonic) potential $U\left(x\right)=2x^{2}$ and a double-well potential $U\left(x\right)=x^{4}-2x^{2}$.

In equilibrium, the Fokker–Planck solution is the Maxwell–Boltzmann distribution, i.e.,

(A7) $$p_{\infty }\left(X,V\right)=\frac{1}{Z}e^{-\beta H(X,V)}=\frac{1}{Z}e^{-\beta \left(\frac{{∥V∥}^{2}}{2}+U\left(X\right)\right)},$$

with partition function $Z=\int e^{-\beta \left(\frac{{∥V∥}^{2}}{2}+U\left(X\right)\right)}dxdv$.

We may compute the energy of each particle at each time point as the sum of its kinetic and potential energies

(A8) $$E_{t}^{\left(i\right)}=\frac{1}{2}{V^{\left(i\right)}}^{2}+U\left(X^{\left(i\right)}\right).$$

Here the superscripts denote individual particles. After the system has reached equilibrium, energy levels per particle are expected to remain constant.

From the equipartition of energy and the fluctuation–dissipation relation, in the long time limit the average kinetic energy of the system is expected to resort to

(A9) $$\lim_{t\to \infty }\langle K\rangle =\lim_{t\to \infty }\frac{1}{2}\langle V^{2}\rangle =\frac{\sigma^{2}}{2\;\gamma }.$$

Symplectic integration [33] of Equation (56) follows the equations

(A10) $$\begin{array}{cc} V_{n+\frac{1}{2}} & =V_{n}+\frac{dt}{2}\left(-\gamma V_{n}+f\left(X_{n}\right)-\frac{\sigma^{2}}{2}\nabla_{v}\ln p_{t}\left(X_{n},V_{n}\right)\right) \end{array}$$

(A11) $$\begin{array}{cc} X_{n+1} & =X_{n}+dt\;V_{n+\frac{1}{2}} \end{array}$$

(A12) $$\begin{array}{cc} V_{n+1} & =V_{n+\frac{1}{2}}+\frac{dt}{2}\left(-\gamma V_{n+\frac{1}{2}}+f\left(X_{n+1}\right)-\frac{\sigma^{2}}{2}\nabla_{v}\ln p_{t}\left(X_{n+1},V_{n+\frac{1}{2}}\right)\right), \end{array}$$

where *n* denotes a single integration step.

### Appendix A.5. Lorenz attractor

For simulating trajectories of the noisy Lorenz system, we employed the following equations

(A13) $$\begin{array}{cc} dx_{t} & =\sigma \left(y-x\right)dt+\sigma dW_{x} \end{array}$$

(A14) $$\begin{array}{cc} dy_{t} & =\left(x(\rho -z)-y\right)dt+\sigma dB_{y} \end{array}$$

(A15) $$\begin{array}{cc} dz_{t} & =\left(x\;y-\beta z\right)dt+\sigma dB_{z}, \end{array}$$

with parameters σ=10, ρ=28, and $\beta =\frac{8}{3}$, that render the deterministic dynamics chaotic [66], employing moderate additive Gaussian noise.

## Appendix B. Computing Central Moment Trajectories for Linear Processes

For a linear process

(A16) $$dX_{t}=A\;X_{t}dt+\sigma dB,$$

the joint density of the state vector *X* remains Gaussian for all times when the initial density is Gaussian. The mean vector *m* and covariance matrix *C* may be computed by solving the ODE system

(A17) $$\begin{array}{c} \frac{dm}{dt}=Am \end{array}$$

(A18) $$\begin{array}{c} \frac{dC}{dt}=AC+CA^{⊤}+\sigma^{2}I. \end{array}$$

## Appendix C. Kullback–Leibler Divergence for Gaussian Distributions

We calculated the KL divergence between the theoretical and simulated distributions with

(A19) $$KL\left(P_{1}\left|\right|P_{2}\right)=\frac{1}{2}\left(\log(|\Sigma_{2}\left|/\right|\Sigma_{1}\left|\right)-d+Tr\left(\Sigma_{2}^{-1}\Sigma_{1}\right)+{\left(\mu_{2}-\mu_{1}\right)}^{T}\Sigma_{2}^{-1}\left(\mu_{2}-\mu_{1}\right)\right),$$

where $P_{x}∼N\left(\mu_{x},\Sigma_{x}\right)$.

## Appendix D. Wasserstein Distance

We employed the 1-Wasserstein distance [59] as a distance metric for comparing pairs of empirical distributions.

For two distributions *P* and *Q*, we denote with J(P,Q) all joint distributions *J* for a pair of random variables (X,Y) with marginals *P* and *Q*. Then the *Wasserstein distance* between these distributions is

(A20) $$W_{p}\left(P,Q\right)={\left(\inf_{J\in J(P,Q)}\int {∥x-y∥}^{p}dJ\left(x,y\right)\right)}^{\frac{1}{p}},$$

where for the 1-Wasserstein distances (used in the present manuscript) p=1.

Interestingly, the Wasserstein distance between two one dimensional distributions *P* and *Q* obtains a closed form solution

(A21) $$W_{p}\left(P,Q\right)={\left(\int_{0}^{1}{∥F_{P}^{-1}\left(\tau \right)-F_{Q}^{-1}\left(\tau \right)∥}^{p}d\tau \right)}^{1/p},$$

with $F_{P}$ and $F_{Q}$ indicating the cumulative distribution functions of P and Q.

Moreover, for one dimensional empirical distributions *P* and *Q* with samples of same size ${\left\{X_{i}\right\}}_{i=1}^{n}$ and ${\left\{Y_{i}\right\}}_{i=1}^{n}$, the Wasserstein distance simplifies into computation of differences of order statistics

(A22) $$W_{p}\left(P,Q\right)={\left(\sum_{i=1}^{n}{∥X_{\left(i\right)}-Y_{\left(i\right)}∥}^{p}\right)}^{\frac{1}{p}},$$

where $X_{\left(i\right)}$ and $Y_{\left(i\right)}$ indicates the *i*-th order statistic of the sample ${\left\{X_{i}\right\}}_{i=1}^{n}$ and ${\left\{Y_{i}\right\}}_{i=1}^{n}$, i.e., $X_{\left(1\right)}\leq X_{\left(2\right)}\leq \cdots \leq X_{\left(n\right)}$ and $Y_{\left(1\right)}\leq Y_{\left(2\right)}\leq \cdots \leq Y_{\left(n\right)}$ [67].

We calculated the average temporal 1-Wasserstein distance as the time average of instantaneous 1-Wasserstein distances between the two distributions under comparison, $P_{t}^{A}$ and $P_{t}^{B}$, i.e.,

(A23) $${\langle W_{1}\left(P_{t}^{A},P_{t}^{B}\right)\rangle }_{t}=\frac{1}{T/dt}\sum_{t=1}^{T/dt}W_{1}\left(P_{t}^{A},P_{t}^{B}\right),$$

where as before *T* stands for the duration of the simulation and dt for the time discretisation step.

## Appendix E. Frobenious Norm

For comparing covariance matrices of the simulated particle systems with ground truth we employed the Frobenious norm of the relevant matrices difference. The Frobenious norm of a m×n matrix *A* may be calculated from

(A24) $${∥A∥}_{F}=\sqrt{\sum_{i=1}^{m}\sum_{j=1}^{n}{\left|a_{i,j}\right|}^{2}}=\sqrt{Tr\left(A^{*}A\right)},$$

where $a_{i,j}$ denote the entries of matrix *A*, and $A^{*}$ stands for the conjugate transpose of A.

## Appendix F. Influence of Hyperparameter Values on the Performance of the Gradient–Log–Density Estimator

To determine the influence of the hyperparameter values on the performance of the gradient–log–density estimator, we systematically evaluated the approximation error of our estimator for N=1000 samples of a one dimensional log–normal distribution with mean μ=0 and standard deviation σ=0.5 for 20 independent realisations.

We quantified the approximation error as the average error between the analytically calculated and predicted gradient-log-density on each sample, i.e.,

(A25) $$\mathrm{Approximation}\;\mathrm{error}=\frac{1}{N}\sum_{i=1}^{N}∥\nabla \ln p\left(x_{i}\right)-\hat{\left(\nabla \ln p\left(x_{i}\right)\right)}∥,$$

where the analytically calculated gradient-log-density was determined as $\nabla \ln p\left(x\right)=\frac{\mu -\sigma^{2}-\ln\left(x\right)}{\sigma^{2}\;x}$.

By systematically varying the regularisation parameter λ, the kernel length scale *l*, and the inducing point number *M* we observed the following:

- The hyperparameter that strongly influences the approximation accuracy is the kernel length scale *l* (Figure A1).
- Underestimation of kernel length scale *l* has stronger impact on approximation accuracy than overestimation (Figure A1).
- For increasing regularisation parameter value λ, underestimation of *l* has less impact on the approximation accuracy (Figure A1 and Figure A2).
- For overestimation of the kernel length scale *l*, the regularisation parameter λ and inducing point number *M* have nearly no effect on the resulting approximation error (Figure A1).
- For underestimation of kernel length scale *l*, increasing the number of inducing points *M* in the estimator results in larger approximation errors (Figure A2 (upper left)).

## Appendix G. Required Number of Particles for Accurate Fokker–Planck Solutions

To compare the computational demands of the deterministic and stochastic particle systems, we determined the required particle number each system needed to attain a specified accuracy compared to ground truth transient solutions. In particular, for a two dimensional Ornstein–Uhlenbeck process, we identified the minimal number of particles $N_{KL}^{*}$ both systems required to achieve a certain time-averaged Kullback–Leibler distance to ground truth transient solutions, ${\langle \mathrm{KL}\left(P_{t}^{A},P_{t}^{N}\right)\rangle }_{t}$. As already indicated in the previous sections, the stochastic system required considerably larger particle number to achieve the same time averaged KL distances to ground truth when compared to our proposed framework. In fact, for the entire range of examined KL distances, our method consistently required at least one order of magnitude less particles compared to the its stochastic counterpart.

## Appendix H. Algorithm for Simulating Deterministic Particle System

Here we provide the algorithm for simulating deterministic particle trajectories according to our proposed framework (Algorithms A1 and A2). In the comments, we denote the computational complexity of each operation in the gradient–log–density estimation in terms of big-O notation. Since the inducing point number *M* employed in the gradient–log–density estimation is considerably smaller than sample number *N*, i.e., M≪N, the overall computational complexity of a *single* gradient-log-density evaluation amounts to $O\left(N\;M^{2}\right)$.

**Algorithm A1:** Gradient Log Density Estimator	
**Input:** *X*: N×D state vector *Z*: M×D inducing points vector *d*: dimension for gradient *l*: RBF Kernel length scale **Output**: *G*: N×1 vector for gradient-log-density at each position *X* in *d* dimension	
**1** $K^{xz}⟵K\left(X,Z;l\right)$	// N×M $O\left(N\;M\right)$
**2** $K^{zz}⟵K\left(Z,Z;l\right)$	//M×M $O\left(M^{2}\right)$
**3** $I\_K^{zz}⟵{\left(K^{zz}+10^{-3}\;I\right)}^{-1}$	// M×M $O\left(M^{3}\right)$
**4** $grad\_K⟵\nabla_{X^{\left(d\right)}}K\left(X,Z;l\right)$	// N×M $O\left(N\;M\right)$
**5** $sgrad\_K⟵\sum_{X_{i}}grad\_K$	// 1×M
**6** $G⟵K^{xz}\;{\left(\lambda \;I+I\_K^{zz}\;{\left(K^{xz}\right)}^{⊺}\;K^{xz}+10^{-3}\;I\right)}^{-1}\;I\_K^{zz}\;sgrad\_K^{⊺}$	// N×1
	// $O\left(N\;M^{2}\right)+O\left(M^{3}\right)$

**Algorithm A2:** Deterministic Particle Simulation
